# Clear phylogeographic pattern and genetic structure of wild boar *Sus scrofa* population in Central and Eastern Europe

**DOI:** 10.1038/s41598-021-88991-1

**Published:** 2021-05-06

**Authors:** Magdalena Niedziałkowska, Ewa Tarnowska, Joanna Ligmanowska, Bogumiła Jędrzejewska, Tomasz Podgórski, Anna Radziszewska, Iwona Ratajczyk, Szilvia Kusza, Aleksei N. Bunevich, Gabriel Danila, Maryna Shkvyria, Tomasz Grzybowski, Marcin Woźniak

**Affiliations:** 1 Mammal Research Institute Polish Academy of Sciences, Białowieża, Poland; 2 Department of Forensic Medicine, Ludwik Rydygier Collegium Medicum in Bydgoszcz, Nicolaus Copernicus University in Toruń, Toruń, Poland; 3 Department of Clinical Pathology, Ludwik Rydygier Collegium Medicum in Bydgoszcz, Nicolaus Copernicus University in Toruń, Toruń, Poland; 4 Department of Game Management and Wildlife Biology, Faculty of Forestry and Wood Sciences, Czech University of Life Sciences, Prague, Czech Republic; 5 Centre for Agrar Genomics and Biotechnology, University of Debrecen, Debrecen, Hungary; 6 State National Park Belovezhskaya Pushcha, Brest Oblast, Kamenec Raion, Kamenyuki, Belarus; 7 Facultatea de Silvicultura, Universitatea Stefan Cel Mare Suceava, Suceava, Romania; 8 Kyiv Zoological Park of National Importance, Kyiv, Ukraine

**Keywords:** Evolution, Genetics, Molecular biology, Zoology

## Abstract

The wild boar *Sus scrofa* is one of the widely spread ungulate species in Europe, yet the origin and genetic structure of the population inhabiting Central and Eastern Europe are not well recognized. We analysed 101 newly obtained sequences of complete mtDNA genomes and 548 D-loop sequences of the species and combined them with previously published data. We identified five phylogenetic clades in Europe with clear phylogeographic pattern. Two of them occurred mainly in western and central part of the continent, while the range of the third clade covered North-Eastern, Central and South-Eastern Europe. The two other clades had rather restricted distribution. In Central Europe, we identified a contact zone of three mtDNA clades. Population genetic structure reflected clear phylogeographic pattern of wild boar in this part of Europe. The contribution of lineages originating from the southern (Dinaric-Balkan) and eastern (northern cost of the Black Sea) areas to the observed phylogeographic pattern of the species in Central and Eastern Europe was larger than those from the regions located in southern France, Iberian, and Italian Peninsulas. The present work was the first mitogenomic analysis conducted in Central and Eastern Europe to study genetic diversity and structure of wild boar population.

## Introduction

Wild boar (*Sus scrofa*) is one of the most numerous ungulates in Europe and the terrestrial mammal species with the widest geographical range^1^. It naturally occurs over large areas in Europe (except for the most northern parts of the continent), Asia and North Africa. The species has also been introduced by humans to all other continents (except Antarctica) and many islands^1^. Wild boar evolved in South-Eastern Asia, and then colonized northern, central and western parts of Eurasia in several migration waves^2,3^. Omnivorous and ecologically flexible, the species occurs in many different temperate and tropical habitats from forests, marshes and grasslands to semi-deserts^1,4^. Furthermore, in many areas of Eurasia wild boar hybridized with domestic pigs^5–7^.

Population numbers of boars have been greatly fluctuating in time. In Europe in the 17-nineteenth centuries, the wild boar number decreased due to climate cooling and overhunting by humans. Since the nineteenth century, their numbers started to increase but then during and after the World Wars in the twentieth century the population was reduced again due to intensive culling. In the second half of twentieth century, the numbers of wild boars significantly increased in Europe^8,9^.

The most important natural factors affecting the fluctuations in population numbers of wild boar are winter temperature, depth of snow and multiannual variation in acorn (oak seed) crop^4,8,10^. At biogeographical scale, the mean January temperature and vegetation productivity index were the best factors explaining the variability in wild boar population densities, whereas predation by wolves *Canis lupus* played a lesser role^11^. Due to high breeding potential, the species is able to recover in numbers very quickly, even when the densities were highly reduced by natural or human-related factors^8^.

Since 2007, when ASF (African swine fever) was introduced to Georgia, the disease has been spreading across Eurasia affecting wild boar and domestic pigs from Western Europe^12^ to Eastern Asia^13,14^. As of 2020, the disease is still expanding, with many European (Bulgaria, Germany, Greece, Hungary, Latvia, Lithuania, Poland, Romania, Serbia, Slovakia) and Asian (China, Hong Kong, North Korea, South Korea, Laos, Vietnam, Myanmar, Cambodia, Indonesia, Philippines, Timor-Leste, Papua New Guinea, India) countries reporting new cases^15^, while only two countries (Czech Republic and Belgium) were declared free of ASF following a successful eradication programme. ASF causes high mortality in wild boar populations resulting in as much as 80% decrease of abundance^16^. Sanitary culling of wild boar, which is one of the preventive measures for ASF control, is further contributing to the decline in the abundance of the species^17,18^.

There have been several phylogenetic studies performed on wild boar in Eurasia^2,3,19–22^, which revealed two or three main mtDNA lineages of wild boar: the Asian (or East Asian and Near Eastern) and the European one divided further into clades. However, the phylogenetic positions of these lineages and clades varied among studies due to different length, number, and geographic distribution of analysed sequences (comp. e.g.,^2,3,20^. The contact zone of the European and the Asian lineages of wild boar occurs in the vicinity of the Caspian Sea in Iran^20^ and the Caucasian Mountains^21^.

Late Pleistocene glaciations, postglacial recolonization, translocations, and hybridization with domestic pigs were pointed out as important factors that shaped the contemporary phylogeographic pattern of wild boar in Europe and western Asia^5,23–25^. The mtDNA studies and localization of subfossil remains indicated that in Europe the species survived the Last Glacial Maximum (LGM) in southern refugia. The mtDNA lineages of wild boar inhabiting Italy, the Caucasus Mountains and the Near East are distinct from all other lineages occurring in western Eurasia^5,26^. Veličković et al.^19^ showed high frequencies of private microsatellite alleles in the populations inhabiting the three southern peninsulas (Iberian, Italian and Balkan), which confirmed the uniqueness of those populations. The more northern areas of Europe have been inhabited by wild boar of admixed origin. They were descendants of specimens recolonizing large areas of Europe from the LGM refugia during the postglacial times^24^. Almost all of them (except several individuals with Asiatic haplotypes, comp.^26,27^) grouped in one mtDNA lineage called E1, which were further divided into clades E1-A and E1-C. The majority of individuals occurring in Central and Eastern Europe belonged to clade E1-C^27^. The genetic structure of that vast population was weak with no signs of significant demographic fluctuations. It was not clear, which of the LGM refugia the contemporary wild boar population in Central and Eastern Europe originated from^27^.

Until now the results of almost all studies concerning the phylogenetic and phylogeographic studies of wild boar in Europe have been based on analyses of relatively short fragments of mtDNA control region (411 bp in^5^; 443 bp in^25^; 637 bp in^24^; 664 bp in^27^). A recently published study by Khederzadeh et al.^26^ for the first time showed the results of phylogenetic analyses of the complete mtDNA genome of wild boar from large areas in North Africa, Europe and Western Asia. The authors evidenced that the Italian E2 lineage of wild boar was closely related to that in the eastern Caucasus Mts., and those lineages were basal to other European haplotypes of the species. Khederzadeh et al.^26^ also suggested that there had been gene flow between those two (Italian and Caucasian) LGM refugial populations and Southern Europe could have been recolonized from the east. This hypothesis has been confirmed by Frantz et al.^23^, who found an extinct mtDNA haplogroup Y2 among Holocene wild boar samples in southern Ukraine (Crimea Peninsula), the Balkans and Italy. That haplogroup was earlier found exclusively among ancient wild boar from Turkey^24^. We hypothesize that also the Eastern and Central parts of European continent were recolonized not only from the well-known southern LGM refugia but also from areas localized in eastern Europe, as it was shown earlier for bank vole *Clethriononys glareolus*^30^, hamster *Cricetus cricetus*^29^ or moose *Alces alces*^30^. To test this hypothesis more detailed analysis of genetic diversity of wild boar population in Central and eastern Europe and western Asia is required.

**Figure 1:**
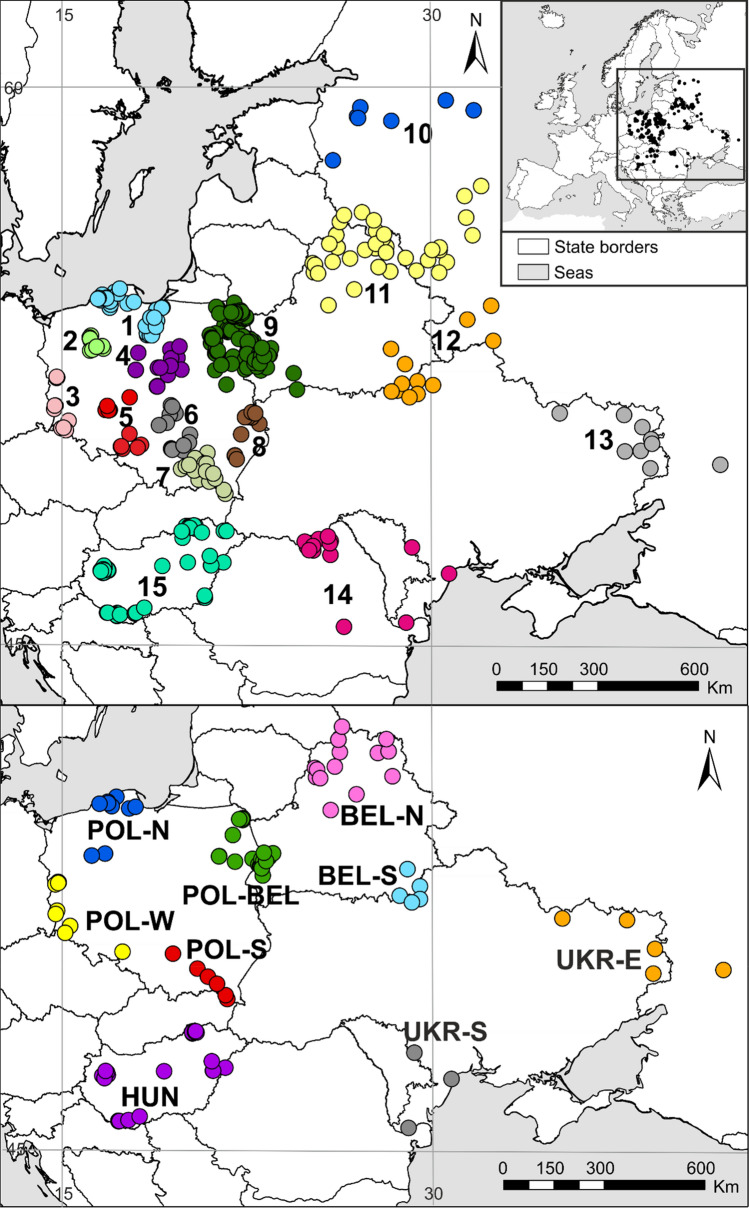
Distribution of wild boar (*Sus scrofa*) samples analysed in this study and grouped into 15 populations for D-loop (control region) analyses (upper panel) and 9 populations for the mitogenome-based analyses (lower panel). The map was created with the ArcGIS software 10.3.1. based on source map from ESRI https://www.esri.com/en-us/home.

In this paper, we report on a study on phylogeny and phylogeography of wild boar in six countries of Central and Eastern Europe, based on 101 sequences of complete mitogenomes and 548 mtDNA control region (D-loop) sequences of the species (Fig. 1, Tables 1, S1). Our data set consist of the largest number of samples and the longest mtDNA sequences analysed in wild boar populations in this part of the continent. To further enlarge the number of analysed sequences and their geographic span, we combined our data with 77 mtDNA sequences published by Khederzadeh et al.^26^. The aims of our study were to: (1) estimate wild boar mitochondrial genetic diversity, (2) reveal their mtDNA phylogenetic and phylogeographic pattern, (3) identify the genetic structure of the species population, and (4) identify the possible regions of origin of the wild boar that currently inhabit Central and Eastern Europe. The analysed samples were collected between 2001 and 2015, thus before the enormous decline of wild boar numbers in Central and Eastern Europe caused by the spread of ASF. Therefore, the results of our study will provide invaluable reference data for future investigations of large scale genetic structure of wild boar population during and after the process of its recovery.

**Table 1:** Number of analysed wild boar tissue samples per country.

Country	Number of samples	
	D-loop	Mitogenomes
Poland	337	39
Belarus	79	30
Ukraine	19	10
Hungary	67	17
Russia	27	2
Romania	19	3
Total	548	101

Russia—European part only. See Fig. 1 for spatial distribution of samples.

## Results

### Genetic diversity, phylogeny and phylogeography of wild boar

**Figure 2:**
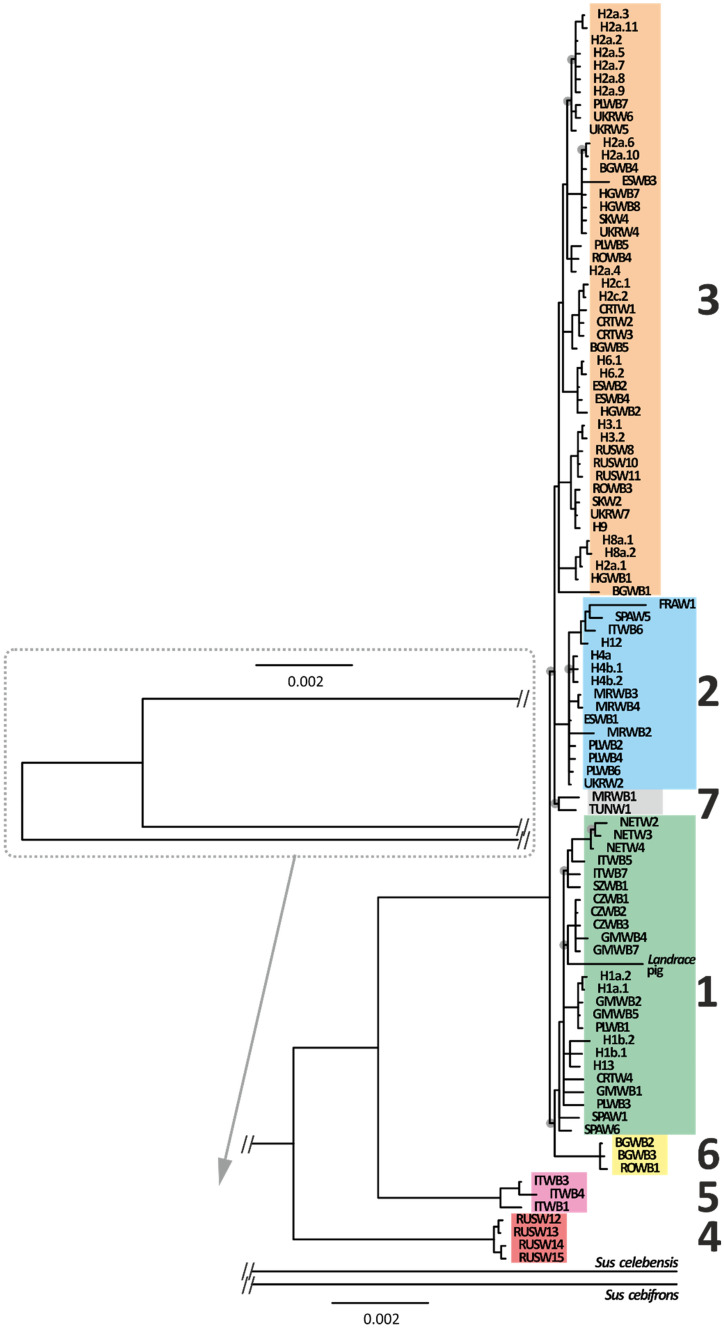
Bayesian phylogenetic tree (MCMC = 1 000,000) of mitogenome sequences of wild boar *Sus scrofa*. Posterior probability values on all branches were between 90 and 100. Black dots mark values in the range 63 – 89.

**Figure 3:**
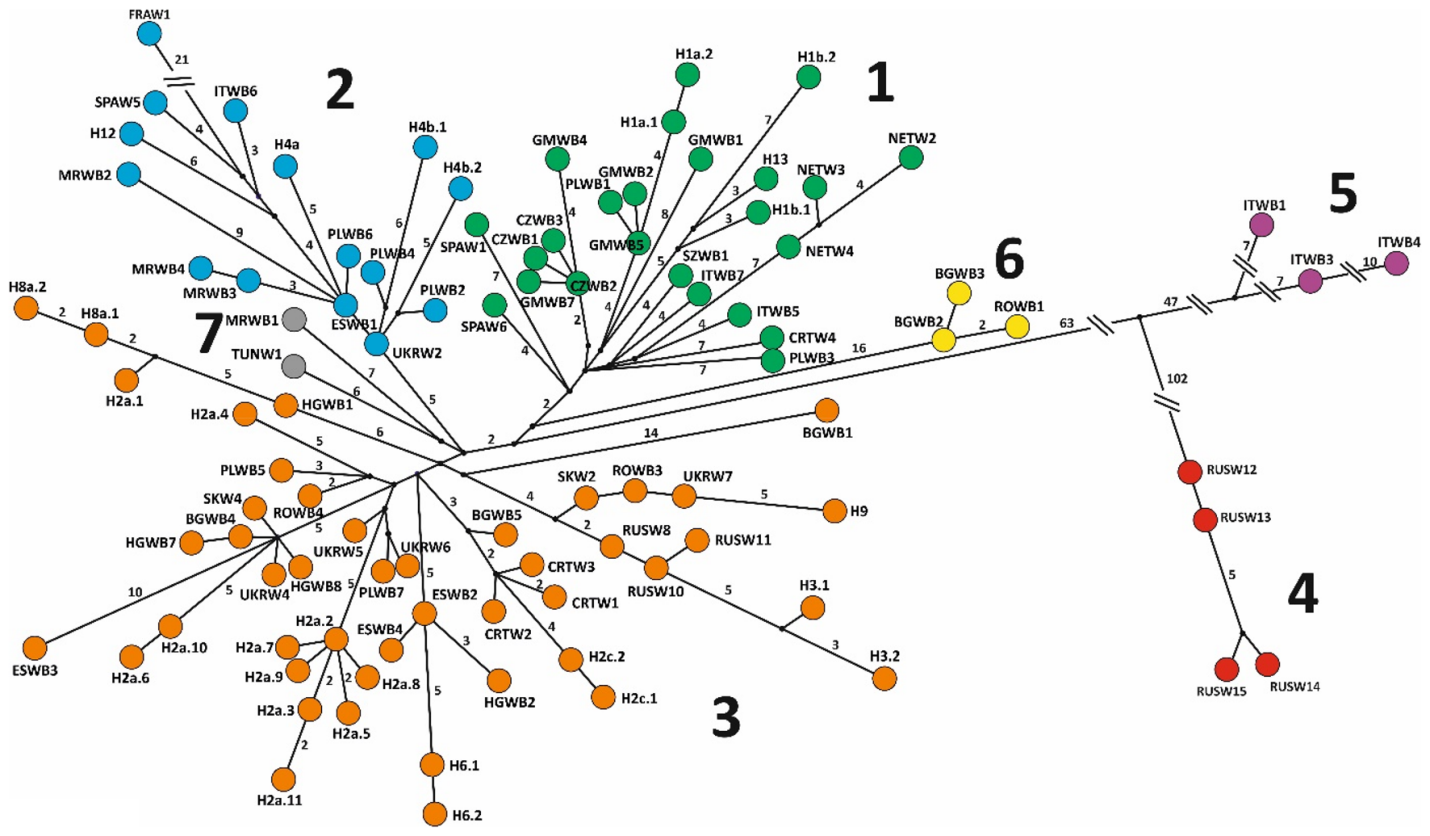
Haplotype network of the mitogenome sequences of wild boar from this study and Khederzadeh et al.^26^. Colours and numbers of clades as in Fig. 2. Numbers on branches mean mutation steps (if > 1) between sequences.

Among 101 sequences of complete mitochondrial genomes of wild boar 29 new haplotypes were identified, not described previously by other authors. Among 548 analysed sequences of mtDNA control region we revealed 19 distinct haplotypes. Based on our analysis of mitochondrial genomes as well as that by Khederzadeh et al.^26^, we identified seven phylogenetic clades of wild boar with high a posteriori probability (> 0.75) (Figs. 2, 3). Clades 5 and 4, which included sequences from Italy and Dagestan (Russia), respectively, were the most distinct genetically (differed by more than 100 mutation steps from all other mtDNA clades, Fig. 3) and represented the oldest evolutionary branches on the tree (Fig. 2). The Italian clade (5) is a sister haplogroup to all the remaining clades of wild boar from Europe and North Africa (Fig. 2). Among these, the most distinct genetically were clade 6 and the haplotype BGWB1 of the clade 3 (Figs. 2, 3), all found in individuals from South-Eastern Europe (Bulgaria and Romania;^26^). The remaining European clades (1–3), and the North African clade 7 differed from each other by 12–15 mutation steps (Fig. 3). The phylogenetic tree based on the D-loop sequences (Fig. S1) showed basically the same structure, yet it slightly differed in topology from the mitochondrial genome tree. As the values of posterior probabilities on its branches were lower, the phylogeny of wild boar based on the whole mitogenomes is more credible.

**Figure 4:**
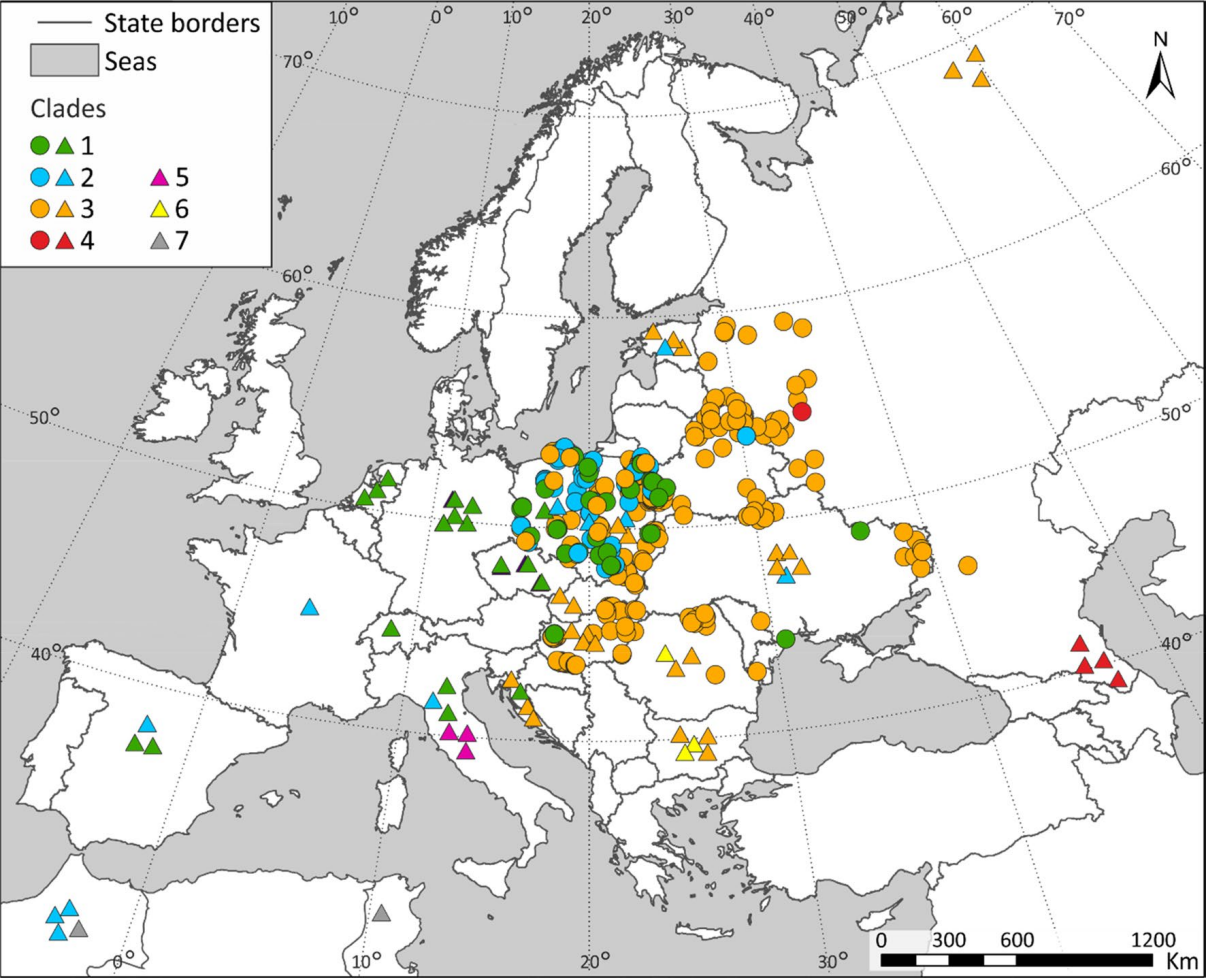
Geographic distribution of clades of wild boar (*Sus scrofa*) distinguished based on the analyses of mtDNA sequences. Colours and numbers of clades as in Fig. 2. Triangles denote mitogenomes published by Khederzadeh et al.^26^, circles—mitogenomic and D-loop sequences obtained in this study. The map was created with the ArcGIS software 10.3.1. based on source map from ESRI https://www.esri.com/en-us/home.

The spatial distribution of the seven clades of wild boar showed a clear phylogeographic pattern in Europe and North Africa (Fig. 4). Clades 1 and 2 occurred mainly in Western and Central Europe. Clade 3 covered the largest area: from the northern Ural Mts. to Eastern, Central and South-Eastern Europe including the Balkans. The remaining clades had more restricted distribution: clade 4 occurred (except one individual from Western Russia) in the Caucasus Mountains, clade 5 in Italy, and clade 6 in South-Eastern Europe (Romania and Bulgaria). Clade 7, although genetically closely related to the European clades 2 and 3, occurred only in the North Africa (Fig. 4). Interestingly, we identified a contact zone of three mtDNA clades: 1, 2 and 3 in the Central Europe, on the territory of Poland (Fig. 4).

### Genetic diversity of wild boar population in Central and Eastern Europe

In our study area the overall haplotype diversity of wild boar mitogenomes was 0.92 (SD = 0.013) and nucleotide diversity 0.0006 (SD = 0.00003) (Table S2). About 0.5% of the nucleotide sites in the sequences were polymorphic. The most numerous haplotypes were H2a.2 (17% of samples) and H6.1 (16%). More than half of the haplotypes were singletons. In the D-loop sequences 2.5% of nucleotide sites were polymorphic. Overall haplotype diversity was 0.71 (SD = 0.017) and nucleotide diversity 0.0015 (SD =  << 0.0001) (Table S4). The most numerous haplotype was H2a (49% of samples). Six haplotypes were singletons.

In Central and Eastern Europe, we detected four mtDNA clades of wild boar, although clade 4 was represented by one individual from western Russia only (Fig. 4). The majority of identified haplotypes of the mitochondrial genome (69%) and the control region (53%) belonged to the most numerous and genetically diverse clade 3 (Table 2). This clade included the most frequent haplotypes of mitochondrial genomes: H2a.2, H3.1 and H6.1 (Fig. S2), which corresponded to the most numerous haplotypes of the control region: H2a, H3 and H6 (Fig. S3). The ranges of the haplotypes H2a and H6 covered the largest area (Fig. S4), although, H6 was rare in Poland and was not found in eastern Ukraine.

**Figure 5:**
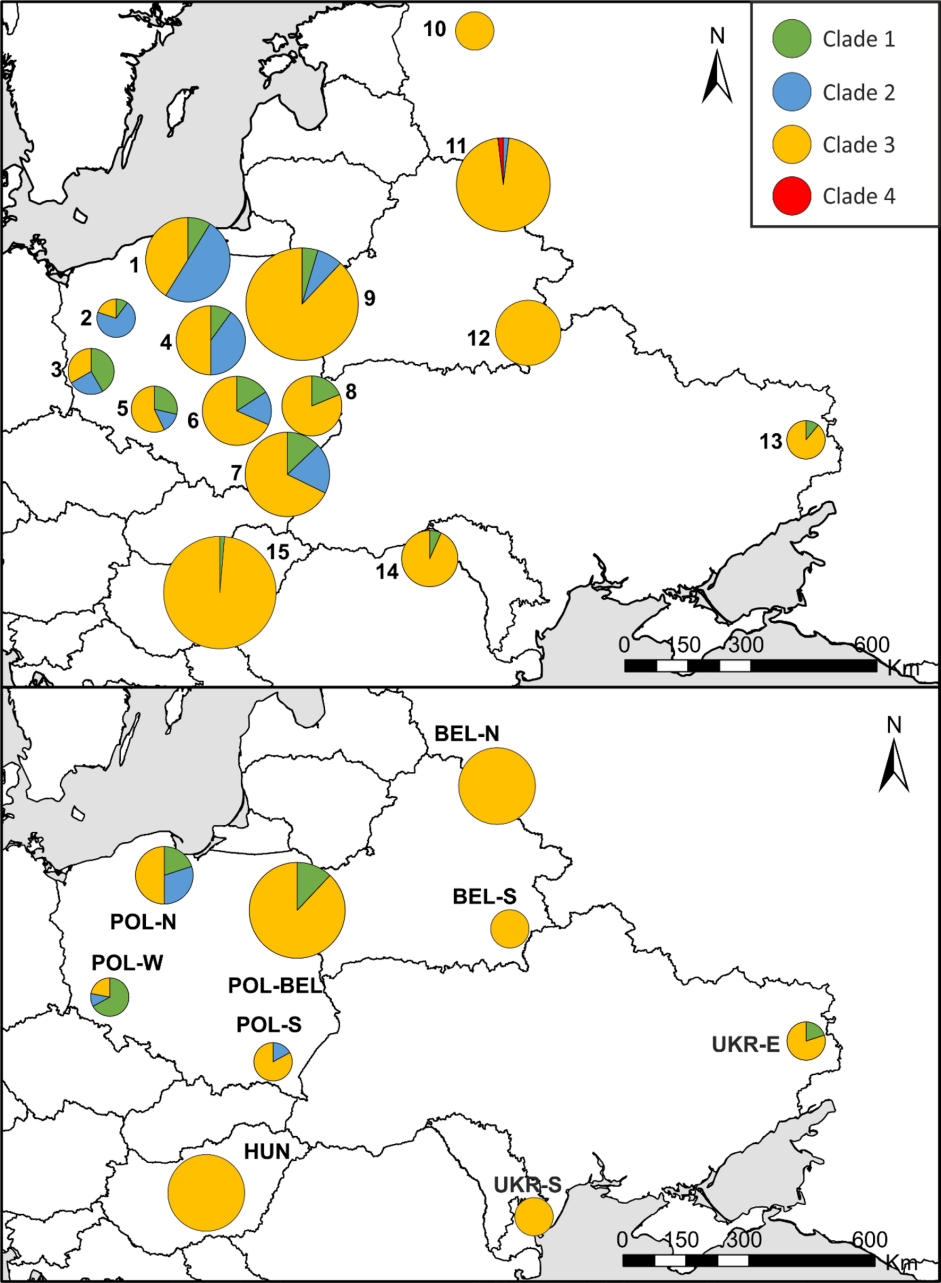
Frequencies of mtDNA clades of wild boar populations based on D-loop (upper panel) and mitogenomic analyses (lower panel). The maps were created with the ArcGIS software 10.3.1. based on source map from ESRI https://www.esri.com/en-us/home.

**Table 2:** Summary statistics of the genetic diversity in three lineages of wild boar in Central and Eastern Europe, based on full mitogenome and D-loop (control region) sequences; *n*—number of individuals, h—number of haplotypes H_d_—haplotype diversity, π × 10^–3^—nucleotide diversity, *B*—haplotype diversity index.

Parameter	Mitogenomes	D-loop
**Clade 1**		
n	13	38
h	5	4
H_d_	0.69	0.36
π × 10^–3^	0.28	0.40
*B*	2.78	1.54
Tajima’s D	− 1.23	− 1.23
Fu’s Fs	2.18	− 1.19
**Clade 2**		
n	5	63
h	4	4
H_d_	0.90	0.50
π × 10^–3^	0.22	0.48
*B*	3.57	1.96
Tajima’s D	− 0.44	− 0.22
Fu’s Fs	− 0.04	− 0.39
**Clade 3**		
n	83	446
h	20	10
π × 10^–3^	0.46	1.05
*B*	7.81	2.35
Tajima’s D	− 0.41	− 0.38
Fu’s Fs	− 0.07	− 1.17

Neutrality tests: Tajimas’s D and Fu’s Fs. Values of neutrality tests were not significant.

The haplotype H3, although widely distributed, did not occur in the southern part of the studied region. Several haplotypes belonging to clade 3 had more restricted distribution, e.g. D-loop haplotype H2c and mitogenomic haplotype H2a.11 occurred only in the southern part of Central and Eastern Europe (Figs S4, S5).

Clades 1 and 2 contributed 17% and 14%, respectively, to the mitogenomic sequences, but 21% each to the D-loop sequences. The nucleotide diversity and *B* values (effective number of haplotypes) of these clades were lower than those of the clade 3 (Table 2). In Central and Eastern Europe haplotypes of clades 1 and 2 occurred mainly in Poland, and most of them were rare and represented only by single individuals (Figs. 5, S4, S5).

Mismatch distribution analyses and neutrality tests (Tajimas’D, Fu’s Fs) performed for the three clades of mitogenomic and D-loop sequences of wild boar did not reveal expansion of those clades (Table 2, Fig. S6).

We compared the frequencies of different mtDNA clades and the indices of genetic diversity among 9 (mitogenomes) and 15 local (D-loop) populations (Fig. 5, Tables S2, S3). The frequency of clade 1 was highest in the South-West Poland (42–67%) and it declined to 20–0% towards south and east (Fig. 5). Clade 2 were found exclusively in Poland, with the exception of one individual recorded in Eastern Belarus. The share of clade 3 was highest in north-eastern, eastern and south eastern parts of Central and Eastern Europe (up to 80–100% in Russia, Belarus, Ukraine and Hungary) and it declined towards west (20–33% in Western Poland).

The haplotype diversity of the mitogenomic populations varied from 0.73 to 1, the values of the *B* index from 3 to 4.76 and the nucleotide diversity ranged between 0.4 and 1 × 10^–3^ (Table S2). The haplotype diversity of the D-loop populations varied from 0.51 to 0.86, the value of *B* index from 1.72 to 5.55 and the nucleotide diversity from 0.7 to 2.0 × 10^–3^. Both indices of haplotype diversity (Hd and B) revealed that the most diverse populations of wild boar in our studied region inhabited southern, central and northern Poland and north-eastern Belarus (Tables S2, S3, comp. Figure 5). The nucleotide diversity of the D-loop populations increased significantly with latitude (r = 0.712, p = 0.003), but we did not find such statistically significant correlations for haplotype diversity indices. The neutrality tests performed for each of the mitogenomic and D-loop populations did not reveal any sings of expansion (Tables S2, S3).

### Genetic structure of wild boar population in Central and Eastern Europe

Genetic differentiation (Fst) between pairs of the analysed populations varied from very low (Fst = 0.001) to high and significant values (0.498), indicating unrestricted gene exchange in the first case and complete isolation in the second one (Tables S4, S5). According to the values of pairwise Fst, the most distant genetically were wild boar from western Poland (populations: mitogenomic POL-W and D-loop pop 2; Tables S4, S5). The Fst values were usually low between pairs of populations located in close proximity (Tables S4, S5). We did not detect significant isolation by distance, as the genetic differentiation did not increase with geographic distance between them (Mantel tests: R = 0.25, p = 0.09 for D-loop and R = 0.04, p = 0.37 for mitogenomic populations).

**Figure 6:**
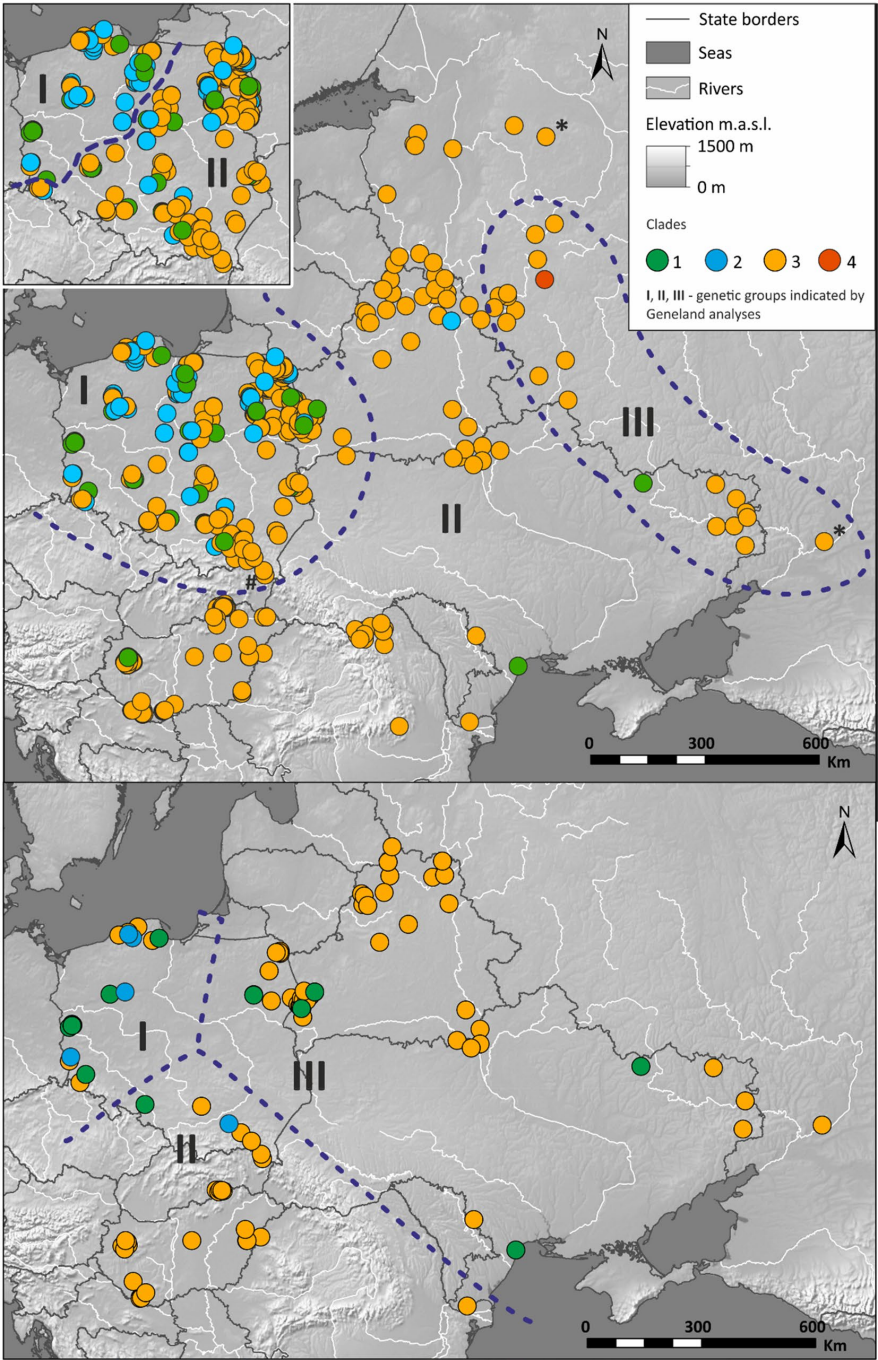
Distribution of three genetic populations indicated by the Geneland software based on the analyses of 548 D-loop sequences (upper panel) and 101 mitogenomes of wild boar (lower panel). Asterik means sequence assigned to one of the genetic populations with 30% probability; # means sequence assigned with 50% probability. All other sequences assigned to their populations with > 50% probability. Insert map in the upper panel: results of Geneland analysis done on D-loop sequences from Poland only. The maps were created with the ArcGIS software 10.3.1. based on source map from ESRI https://www.esri.com/en-us/home.

Analyses performed using Geneland for both D-loop and mitogenomic sequences showed three genetic groups of wild boar in Central and Eastern Europe, although the distribution of these groups were different for each set of the populations (Fig. 6). D-loop sequences were assembled in three groups GI – GIII and mitogenomic sequences in groups GmI–GmIII (Fig. 6). D-loop sequences were clumped into GI consisting of samples from Poland and a few from western Belarus and western Ukraine, GII covering north-western Russia, Belarus, northern and southern Ukraine, Romania and Hungary and GIII grouping the easternmost samples from eastern Ukraine and western Russia (Fig. 6). However, if Geneland was run on D-loop sequences from Poland only, the border between two identified genetic groups was very similar to that between mitogenomic groups GmI and GmIII (see insert in Fig. 6, upper panel). Based on mitogenomic sequences, Geneland separated three groups: GmI in north-western Poland, Gm II in southern Poland, Slovakia, Hungary and the southern-most Ukraine, and GmIII in north-eastern Poland, Belarus, Ukraine and western Russia (Fig. 6).

Among the D-loop genetic groups the highest haplotype diversity was detected in the GII group and nucleotide diversity was the highest in the GIII group (Table S6). The highest haplotype diversity among three mitogenomic genetic groups was observed in the GmI group, and nucleotide diversity in the GmIII group (Table S6). The Fst values calculated among all pairs of genetic groups (D-loop and mitogenomic) were statistically significant in all cases but the largest genetic differentiation was detected between mitogenomic groups GmI and GmII (Fst = 0.22, Table S7) and the lowest between D-loop groups GI and GII (Fst = 0.08, Table S8).

## Discussion

Previous studies on European wild boar, based on shorter fragments of mtDNA (from 80 diagnostic bp in^2^, to 410–660 bp in Scandura et al.^5^ and Kusza et al.^27^, respectively) did not show any clear phylogeographic structure within the large pan-European clade E1, spanning from Iberia to Ukraine and western Russia. The proposed division of E1 into A and C clusters^2,23^ soon appeared too simplified, as several regional studies revealed numerous subclades of intermediate phylogenetic position between A and C and geographically restricted ranges of occurrence, namely in Greece^24^ and the Dinaric-Balkan region (from Slovenia to North Macedonia^25^). This work, based on 1175-bp long fragment of mtDNA and full mitogenomes of wild boar (from our study combined with those published by Khederzadeh et al.^26^) described the phylogeny of wild boar with much higher resolution and allowed for the spatial lineage sorting across Europe.

We found seven phylogenetic clades of wild boar in the geographic area from North Africa, to the mainland of Europe, to Dagestan in the North Caucasus. We confirmed the findings by Khederzadeh et al.^26^ that haplotypes from Dagestan and Italy (our clades 4 and 5, respectively) were genetically most distinct from all other lineages in Europe. Our clade 5 corresponded to E2 and clades 1–3, 6 and 7 (the last one found in North Africa) to the pan-European clade E1^2,5,31^. Clade 6, not reported in earlier studies, with 3 haplotypes from Bulgaria and Romania, along with the most distinct haplotype BGWB1 from Bulgaria (clade 3) are most likely signals of very diverse populations in the Balkans (comp.^24,25^). This requires further detailed studies including more dense sampling in that region.

Clades 1 and 2 occurred widely in western and central Europe (from Iberia to Czech Republic and Poland) and only sporadically further south and east, whereas clade 3 appeared and increased in frequency to be a dominant one among wild boar populations in Eastern Poland, Hungary, Belarus, Ukraine, Romania and Western Russia. Therefore, a complete exchange of clades takes place between the two longitudinal extremes of continental Europe. A contact zone of one eastern and two western clades (about 700 km wide in Poland) probably runs diagonally from Croatia, Western Hungary, to Poland and the Baltic States. Our dense sampling in Central and Eastern Europe, where the eastern clade 3 was dominating, revealed—not surprisingly—that it was genetically diverse, with further spatial substructuring: groups of haplotypes with different positions on the branches of the phylogenetic trees and networks had different frequencies regionally. Future research should focus on a comparable dense sampling of wild boar from western and southern Europe for mitogenomic analyses. This will be essential to reveal the spatial pattern of clade 1 and 2 frequencies in Iberia, Italy, and Western Europe.

In Central and Eastern Europe, the highest genetic diversity was detected in the contact zone of different mtDNA clades that occurred in wild boar populations inhabiting the Western, South-Western, and Central Poland. According to the obtained Fst values and distribution of different haplotypes, the most intensive gene flow among wild boar populations in our study area is in the north-east to south-east direction and the east–west direction. Wild boar inhabiting Western Poland were genetically most distant among the analysed populations. This is in agreement with the results of Veličković et al.^19^, who showed that populations of wild boar with admixture origin had larger genetic diversity than the refugial populations. The identification of the easternmost subpopulation, indicated by the analysis of D-loop sequences in Geneland, was in line with the earlier results of Kusza et al.^27^. Further sampling of wild boar populations in the European part of Russia, especially in the gap between Eastern Ukraine and the Caucasus Mountains might reveal another contact zone of clades 3 and 4 and possibly the Asiatic clades. Such a contact zone of European, Near East and Asiatic clades of wild boar was found south of the Caucasus Mts., in Northwestern Iran^20,23^.

The clear phylogeographic patterns of the identified clades 1–6 raises a question about the glacial refugia of wild boar, and the contributions of the refugial populations to the contemporary gene pool of the species in Europe. The contact zones of different genetic lineages in Central and Eastern Europe were identified for several other mammalian species and they testify to the origin of those lineages from different refugia (e.g.^32–34^). According to fossil records^35^, during the LGM wild boar inhabited Northern Spain, Central Portugal, Southern France, South-central and North-western Italy, Slovenia, Croatia, and Greece. Sommer and Nadachowski^35^ did not analyse the eastern regions, such as Black Sea coasts and the Caucasus. Reconstruction of wild boar LGM range by modelling based on the present-time habitat suitability of the species was done by Vilaça et al.^36^, however, this did not include Russian populations. Thus, the model of LGM refugia only produced three candidate regions: Iberia-Southern France, Italy, and Greece. Another approach was applied by Markova and Puzachenko^37^, who reconstructed the ecosystems of Europe (the whole continent from the Atlantic and Mediterranean coasts to the Ural Mountains) based on records of indicative plant (spores and pollen) and mammal (fossils) species. Habitats suitable for wild boar (Mediterranean forest, Caucasian forest, and the southern variant of periglacial forest-steppe) were supposed to form the continuous belt of variable width covering northern half of the Iberian peninsula, southern France, Apennine peninsula (without its southern part) up to the Alps, the whole Balkan region (with the exception of the southernmost Peloponnese peninsula), the Pannonian Basin, the northern and eastern coast of the Black Sea, ending around the Caucasus Mountain ridge, with a larger refugial area on its southern side [37 p. 46]. Ecological niche modelling performed by Niedziałkowska et al.^38^ for the European red deer (*Cervus elaphus*), a species with habitat preferences similar to those of wild boar, also showed that the surroundings of the Black Sea provided suitable refugium for deer during LGM.

Interestingly, phylogeographic studies on the present-day wild boar populations inhabiting their presumed LGM refugia evidenced great genetic diversity in those regions, and the endemic character of the local gene pools rather than source of postglacial recolonization of Europe. This was true for E2 clade in Italy^2^, Southern Balkan haplogroup^25^, numerous haplotypes from Greece and Spain^24^. The last authors concluded that those southernmost peninsular regions still harbour the remnants of pre-LGM gene pool, yet they played no role in the recolonization of Central-Eastern Europe. The recolonization must have been only based on the gene pool in the northern parts of the refugia (leading-edge colonization;^39^).

Vilaça et al.^36^ believed that the Iberian refugium did not significantly contribute to the post-glacial European range of wild boar, and proposed two major colonization routes: one starting from the refugium in southern France and/or northern Italy, and leading wild boars to western and central Europe, and the second one starting from the Balkans and reaching north-eastern parts of the continent. Veličković et al.^25^ argued that mtDNA lineages originating from Iberia, Italian and Balkan Peninsulas played a similar role in the post-glacial recolonization of Europe by wild boar. The results of our study partly confirmed the hypotheses of Vilaça et al.^36^ and Veličković et al.^25^. It is highly probable that clades 1 and 2 spread from south-west towards central and eastern Europe, whereas wild boar belonging to clade 3 could have arrived from the south and/or south-east and populated the central and north-eastern Europe. The two colonization waves met in central-eastern part of the continent and formed a contact zone. As regards clade 3, we suppose that the LGM refugium of this highly diverse clade covered the Dinaric and the Balkan region as well as the contiguous northern coast of the Black Sea. As suggested by the spatial distribution of subgroups of related haplotypes (comp. Figs S4 and S5), the northward dispersal of wild boar in the postglacial period began both from the south (Dinaric-Balkan) and the south-east (Black Sea northern coast).

To answer the question where the western waves of colonizers (clades 1 and 2) originated from, we would need to expand the sampling and mitogenome analyses of wild boar in the gaps between the area of the contact zone and the potential refugial regions in Iberia, France, and Italy.

Although the genetic diversity of wild boar populations in Europe was higher in the past, than nowadays, the spatial distribution of the main mtDNA clades (E1, E2 and the Near Eastern) has not changed significantly since the early Holocene, in contrast to the mtDNA phylogeographic pattern of the domestic pigs^21,23,40^. Only one clade of the species (Y2 according to nomenclature provided in^21,22^), occurring in the past in southern and south-eastern Europe but genetically rather closely related to the Near Eastern wild boar [21, this study Figs S7, S8], is today extinct. Furthermore, according to Scandura et al.^23^, hybridization with pigs has not had any significant impact on the genetic diversity of wild boar.

Despite fluctuations in wild boar numbers in the recent past (e.g. population decrease in the Little Ice Age of the eighteenth-nineteenth centuries), the species never disappeared from Central and Eastern Europe^8^. Therefore, such climate-driven oscillations probably did not have significant impact on the phylogeographic pattern of wild boar. A similar situation was recorded in another temperate ungulate species—the European red deer. Although the genetic diversity of deer was higher in the past and their numbers significantly decreased or they even became extinct in the eighteenth–nineteenth centuries in some regions of north-central Europe, the present-day phylogeographic pattern of the species reflects the postglacial migration routes from different LGM refugia localized in western and south-eastern parts of Europe (e.g.^32,41–43^). Further studies on wild boar genetics including also nuclear DNA markers are needed to confirm the phylogenetic pattern of wild boar in this part of Europe. Nonetheless, the study performed by Frantz et al.^21^ suggested that, on a larger spatial scale, the phylogeographic pattern obtained based on the mtDNA data and whole genomes were concordant.

## Conclusions

Analyses of the whole mitogenomes of wild boar and combination of the obtained sequences with previously published data revealed the phylogeny of European wild boar with higher resolution. We detected five European clades of the species with clear phylogeographic pattern. During the post-glacial times, Central and Eastern Europe was probably recolonized by wild boar belonging to at least two western clades and one large, diverse clade from south-eastern Europe. Nowadays, the contribution of mtDNA lineages originating from the south-east (most probably the Dinaric-Balkan region and the contiguous area of northern coast of the Black Sea) to the wild boar population in Central and Eastern Europe, is larger than that of clades originating from western and south-western part of the continent. Genetic diversity was largest in the populations occurring in the contact zone of three different clades of wild boar. Genetic differentiation (Fst) among studied populations varied from very low to very high values. No isolation by distance was detected. The most intensive gene flow among studied wild boar populations took place in the north-east to south-east direction and in the east–west direction. Population genetic structure of wild boar in Central and Eastern Europe reflected the phylogeographic pattern of this species.

## Material and methods

### Collection and laboratory analyses of samples

We analysed tissue samples and hair follicles of 548 wild boars collected in six countries of Central and Eastern Europe in 2001–2015 (Table 1). Tissue samples were provided by licensed hunters. Hair follicle samples came from the zoological collection of the Mammal Research Institute Polish Academy of Sciences in Białowieża, Poland. Ethic permissions were not required. No animals were killed or hurt for the purposes of this project. The study did not involve live animals.

We sequenced mitochondrial control region (D-loop of 1175 bp) of all individuals and full mitogenome (16,615 bp) of selected (according to their localizations in different areas) 101 individuals (Table 1). We divided the analysed 548 wild boar into 15 populations, and the 101 individuals with sequenced mitogenomes into 9 populations according to their sampling regions (Fig. 1). A list of sampling localities with the exact number of specimens and years of sampling is given in Table S1.

The whole genomic DNA was extracted using BioTrace DNA Purification Kit (Eurx, Poland). The sequence of mitochondrial control region (1175 bp) was amplified using two sets of primers: (1) SSmtDNApm33F 5′-ATACCAATCACTAGCATCATCG-3′; SSmtDNApm34R 5′ GAGTTCCATGAAGTCCAGCTAC-3′^44^ and (2) 1F: 5′ CTTACTTCAGGACCATCTCA-3′; 1B: 5″- GGTTGAGCAAGGCGTTAT-3′^45^. Full mitochondrial genome was amplified using 19 sets of primers described by Jiang et al.^45^ (Table S9). PCR reactions were set in 25 µl volume and run in 2720 Thermal Cycler (Applied Biosystems, US). Reaction mix contained GoTaq Hot Start Polymerase (Promega, US) 1 × buffer, 1.5 mM MgCl_2_, 250 µM dNTP’s, 2 × 10 pmol of primers, 0.5 U Taq polymerase, and 0.3 µl DNA. PCR profile consisted of initial denaturation step at 94 °C for 5 min followed by 32 cycles of 94 °C for 30 s, 55 °C for 30 s, and 68 °C for 90 s, with final elongation step at 68 °C for 10 min. PCR products were checked on 3% agarose gel with addition of ethidium bromide and were purified using Amicon Ultra – 0.5 Centrifugal Filter Devices (Millipore, Germany). Next, we used both primers (forward and reverse in separate sequencing reactions for each pair of primers) and BigDye Terminator (BDT) v3.1 Cycle Sequencing Kit (Applied Biosystems, US) to obtain sequences of the control region and mitogenome from both strands of each DNA fragment. The sequencing reaction mix contained 3 µl 5X BDT buffer, 1 µl BDT RR Mix, 0.32 µl (10 pmol concentration) primer, 3 µl of amplified and purified DNA and 2.68 µl of distilled water. Sequencing PCR profile consisted of initial denaturation step at 96 °C for 1 min followed by 30 cycles of 96 °C for 10 s, 50 °C for 5 s, 60 °C for 4 s. Sequencing products were precipitated and purified using mix of 96% ethanol, 3 M sodium acetate and distilled water (exact description of precipitation process is given in Supplementary Materials). The obtained pellet was suspended in 30 µl of HI-DI. 10 µl of suspended sequencing product was placed on the 96-well plate, denatured at 94 °C for 3 min, then cooled on ice for 3 min and analysed on 3130xl GeneticAnalyzer (Applied Biosystems, US). Sequences were checked and aligned to the reference sequence (GenBank accession number AF034253) using SeqScape v. 2.5 software (Applied Biosystems, US).

### Statistical analysis

The phylogenetic relationships among wild boars, both for mitogenomic and D-loop sequences, were reconstructed using Bayesian phylogenetic tree in MrBayes 3.2.7 software^46^. HKY + G + I evolutionary model was used after the model selection in Mega 7.0.26^47^. Two independent Monte Carlo Markov Chains (MCMC) were run for 10,000,000 generations, with samplefreq = 100, printfreq = 500, diagnfreq = 5000, standard deviation after 10,000,000 mcmc < 0.005). Phylogenetic trees were visualised in FigTree 1.4.4 (http://tree.bio.ed.ac.uk/software/figtree/). For phylogenetic comparisons, the sequences obtained in this study were combined with those from^26^ pre-processed in SAMtools^48^ to reconstruct full nucleotide sequences. Trees were rooted with sequences of two swine species: *Sus cebifrons* and *Sus celebensis* (GenBank accession numbers: KF952600 and KM203891). Phylogenetic network for mitogenomic haplotypes from both studies (our and those of Khederzadeh et al.^26^) were constructed with Maximun likelihood (ML) approach using Phylip 3.695 (http://evolution.genetics.washington.edu/phylip.html) and Haplotype Viewer (http://www.cibiv.at/~greg/haplo-viewer). The networks for all sequences obtained in this study for the two markers (mitogenomes and D-loop) were also constructed using Median joining method in PopArt 1.7^49^.

Parameters of genetic diversity (number of haplotypes, number of polymorphic loci, and haplotype and nucleotide diversity) were calculated for the two mtDNA markers in DNAsp 6.11.01^50^. We also calculated B index using the formula B = 1/Σp*i*^2^ where p*i* is the frequency of haplotype *i* in the population^51^. It ranges from 1 to *n*—where *n* is the total number of detected haplotypes. This index better reflects the effective number of haplotypes in a sample due to its wider range than the commonly used haplotype diversity index (see^28,33^). Two tests of neutrality (Tajima’s D and Fu’s Fs) as well as their statistical significance for populations and for whole sample set in the two markers were calculated in DNAsp. We checked the Isolation by Distance (IBD) for both markers using Mantel Test performed in Arlequin v3.5.2.2^52^. The described parameters of genetic variability were also calculated for each of the three main mtDNA clades of wild boar detected in Central and Eastern Europe. Neutrality tests, using DNAsp, and mismatch analysis, using Arlequin, were also performed for each of the clades detected by mitogenome and D-loop sequences.

Correlations between genetic variability parameters (haplotype diversity, nucleotide diversity, B index), coordinates of the localities of mitogenomic or D-loop populations and sample size were calculated using the *rcor.test* implemented in “ltm” package R (Rizopoulos 2006, https://www.r-project.org/).

To detect the genetic structure of wild boars, we ran three independent Geneland ver. 4.9.2^53–58^ analyses: for mitogenomes, for all D-loop sequences, and for D-loop sequences only from Poland, were performed under spatial model with 100,000 iterations, thinning = 100, and 20 multiple independent runs. Parameters of genetic diversity for the groups indicated by Geneland were calculated using the software described above and used for calculation of such variables for populations. To evaluate gene flow, Fst values among all pairs of populations (for the two markers) and genetic groups indicated by Geneland ver. 4.9.2^53–58^ were calculated in Arlequin.

## Supplementary Information

Supplementary Information.

Supplementary Information.

## Data Availability

D-loop sequences obtained in this study are deposited in GenBank (Accession numbers: MW842550-MW842568). Other datasets generated during and/or analysed during the current study are available from the corresponding author on reasonable request.
