# Supplementary material for: Clear phylogeographic pattern and genetic structure of wild boar Sus scrofa population in Central and Eastern Europe
Source: Sci Rep. 2021 May 6;11:9680. doi: 10.1038/s41598-021-88991-1 (PMC8102581; doi:10.1038/s41598-021-88991-1)
Supplement: Supplementary file 1 — Supplementary Information. [file 41598_2021_88991_MOESM1_ESM.docx]

**Supplementary Materials**

**Clear phylogeographic pattern and genetic structure of wild boar *Sus scrofa* population in Central and Eastern Europe**

**Magdalena Niedziałkowska^1^*, Ewa Tarnowska^1^, Joanna Ligmanowska^2,3^, Bogumiła Jędrzejewska^1^, Tomasz Podgórski^1,4^, Anna Radziszewska^2^, Iwona Ratajczyk^2^, Szilvia Kusza^5^, Aleksei N. Bunevich^6^, Gabriel Danila^7^, Maryna Shkvyria^8^, Tomasz Grzybowski^2^, Marcin Woźniak^2^**

*Corresponding author: email: [mniedz@ibs.bialowieza.pl](mailto:mniedz@ibs.bialowieza.pl)

^1^Mammal Research Institute Polish Academy of Science, Białowieża, Poland

^2^ Department of Forensic Medicine, Ludwik Rydygier Collegium Medicum in Bydgoszcz, Nicolaus Copernicus University in Toruń, Poland

^3^ Department of Clinical Pathology, Ludwik Rydygier Collegium Medicum in Bydgoszcz, Nicolaus Copernicus University in Toruń, Poland

Anna Radziszewska: [anna.radziszewska@cm.umk.pl](mailto:anna.radziszewska@cm.umk.pl)
Iwona Ratajczyk::[milewskaiwona@wp.pl](mailto:milewskaiwona@wp.pl)
Tomasz Grzybowski: [tgrzyb@cm.umk.pl](mailto:tgrzyb@cm.umk.pl)

^4^ Department of Game Management and Wildlife Biology, Faculty of Forestry and Wood Sciences, Czech University of Life Sciences, Prague, Czech Republic

^5^ Centre for Agrar Genomics and Biotechnology, University of Debrecen, Hungary

^6^ State National Park Belovezhskaya Pushcha, Brest Oblast, Kamenec Raion, Kamenyuki, Belarus

^7^ Universitatea Stefan cel Mare Suceava, Facultatea de Silvicultura, Romania

^8^ Kyiv Zoological Park of National Importance, Kyiv, Ukraine

**Precipitation and purification of sequencing products**

10 µl of the sequencing PCR product was added to the mix consisted of 62.5 µl 96% ethanol, 3 µl 3 M sodium acetate and 14.5 µl distilled water. Next, it was incubated at room temperature for 15 min and centrifuged with 14 000 rpm for 20 min at 4^o^C in vacuum centrifuge Concentration 5301 (Eppendorf). Supernatant was removed, 250 µl of 70% ethanol was added to the mix and it was centrifuged with 14 000 rpm for 10 min at 4^o^C. Finally, supernatant was removed and the obtained pellet was suspended in 30 µl of HI-DI.


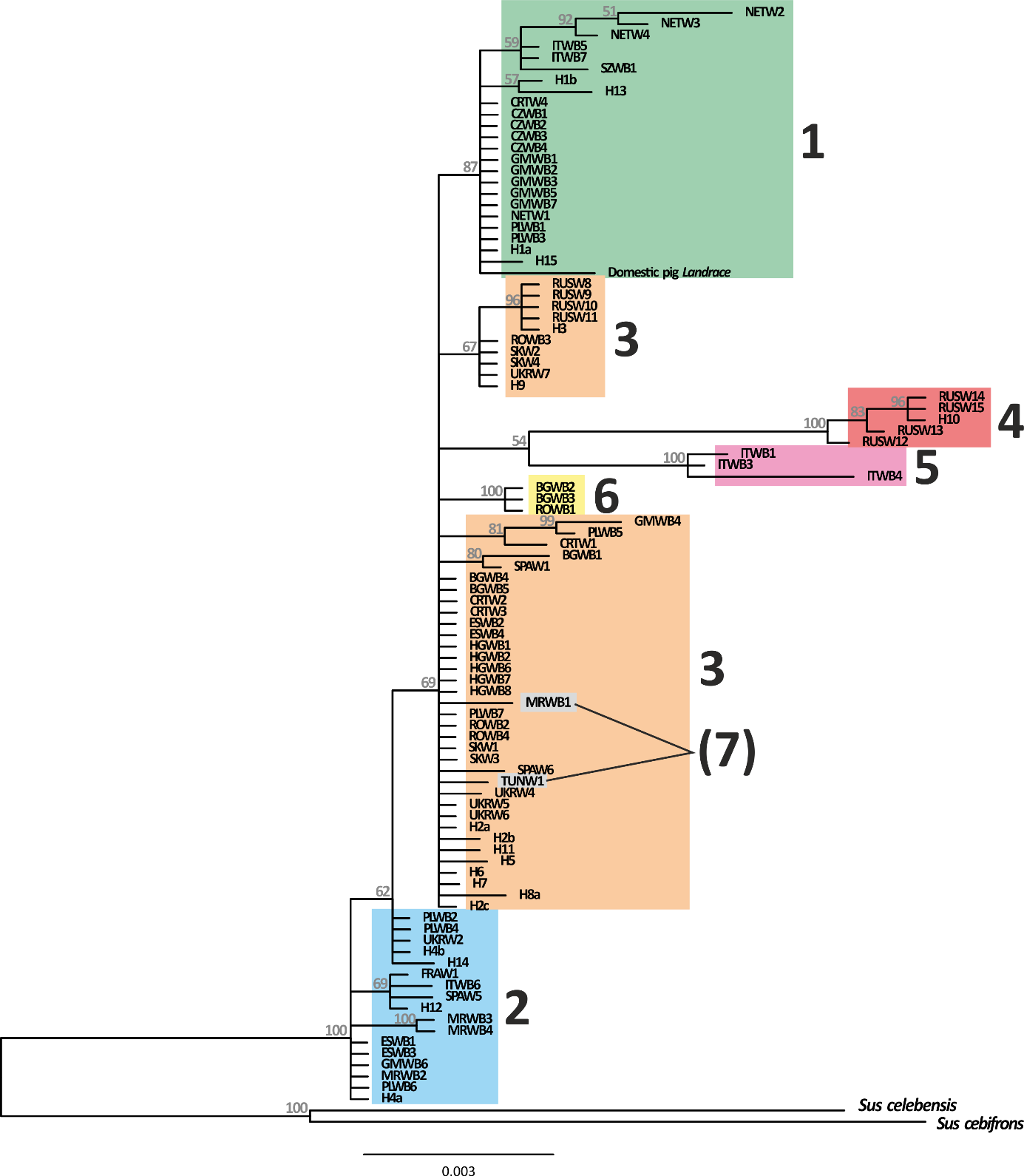


Fig. S1. The Bayesian phylogenetic tree (MCMC = 1 000 000) of D-loop sequences of wild boar *Sus scrofa*. Colours and numbers of clades as in Fig. 2; (7) – denotes haplotypes that were distinguished as Clade 7 by mitogenome analysis. Number on the branches indicate posterior probability values.


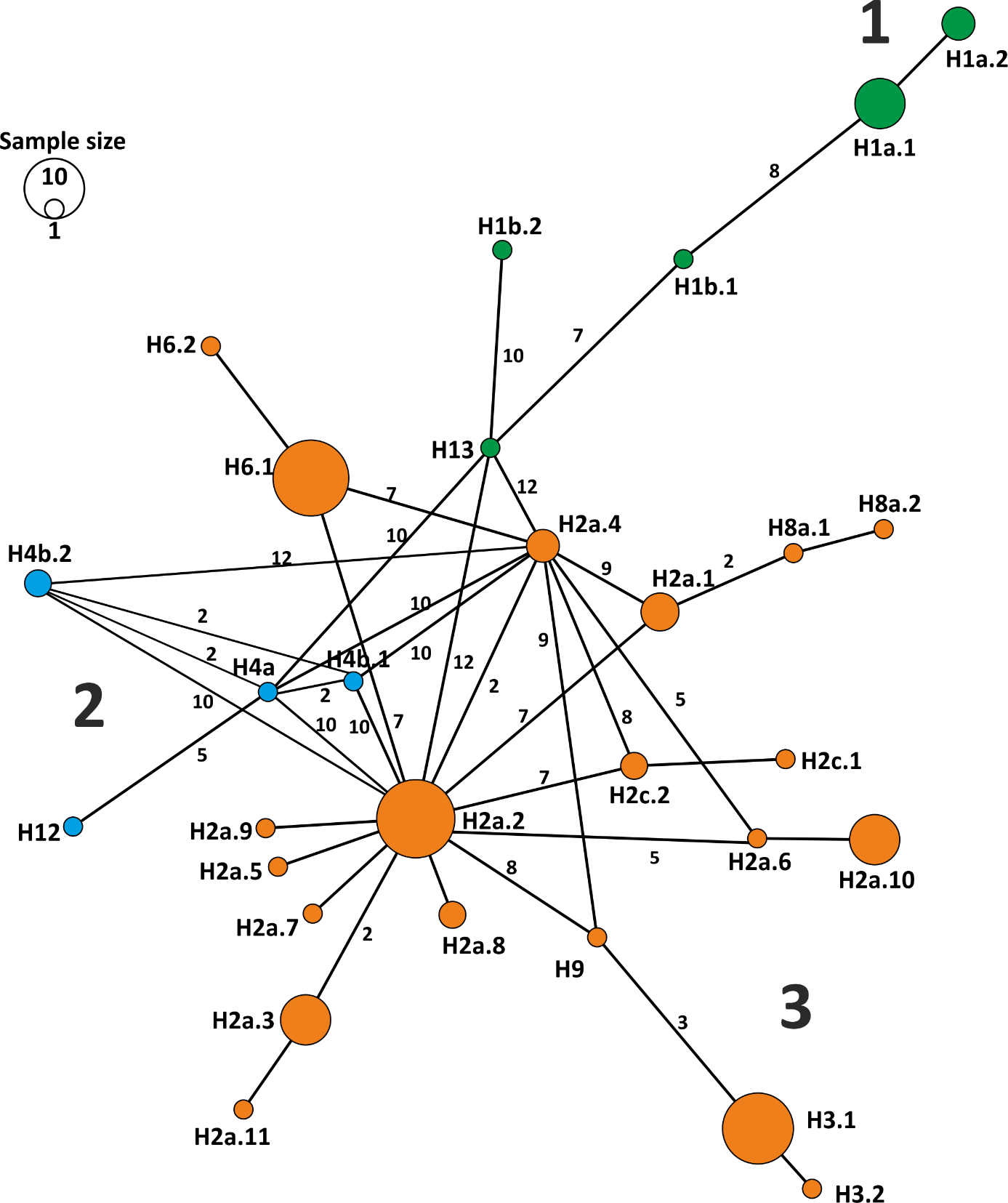


Fig. S2. Haplotype network of the mitogenome sequences of wild boar (*Sus scrofa*) obtained in this study. Colours and numbers of clades as in Figs 2 and S1. Numbers on branches represent mutation steps between sequences (if > 1). The size of circle correspond to the numbers of samples with a given haplotype.


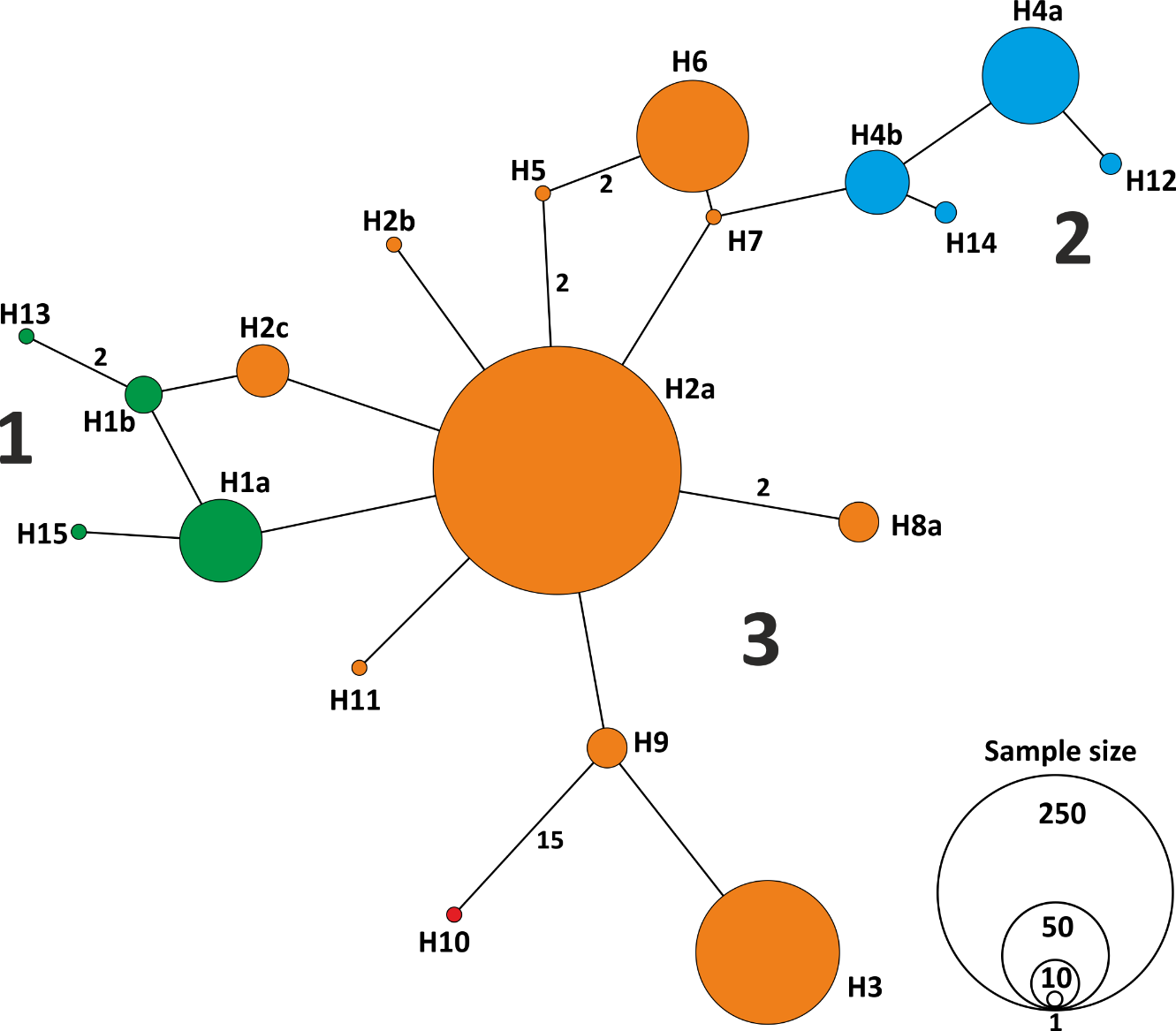


Fig. S3. Haplotype network of D-loop sequences of wild boar (*Sus scrofa*) obtained in this study. Colours and numbers of clades as in Figs 2 and S1. Numbers on branches represent mutation steps between sequences (if > 1). The size of circle corresponds to the number of samples with a given haplotype.


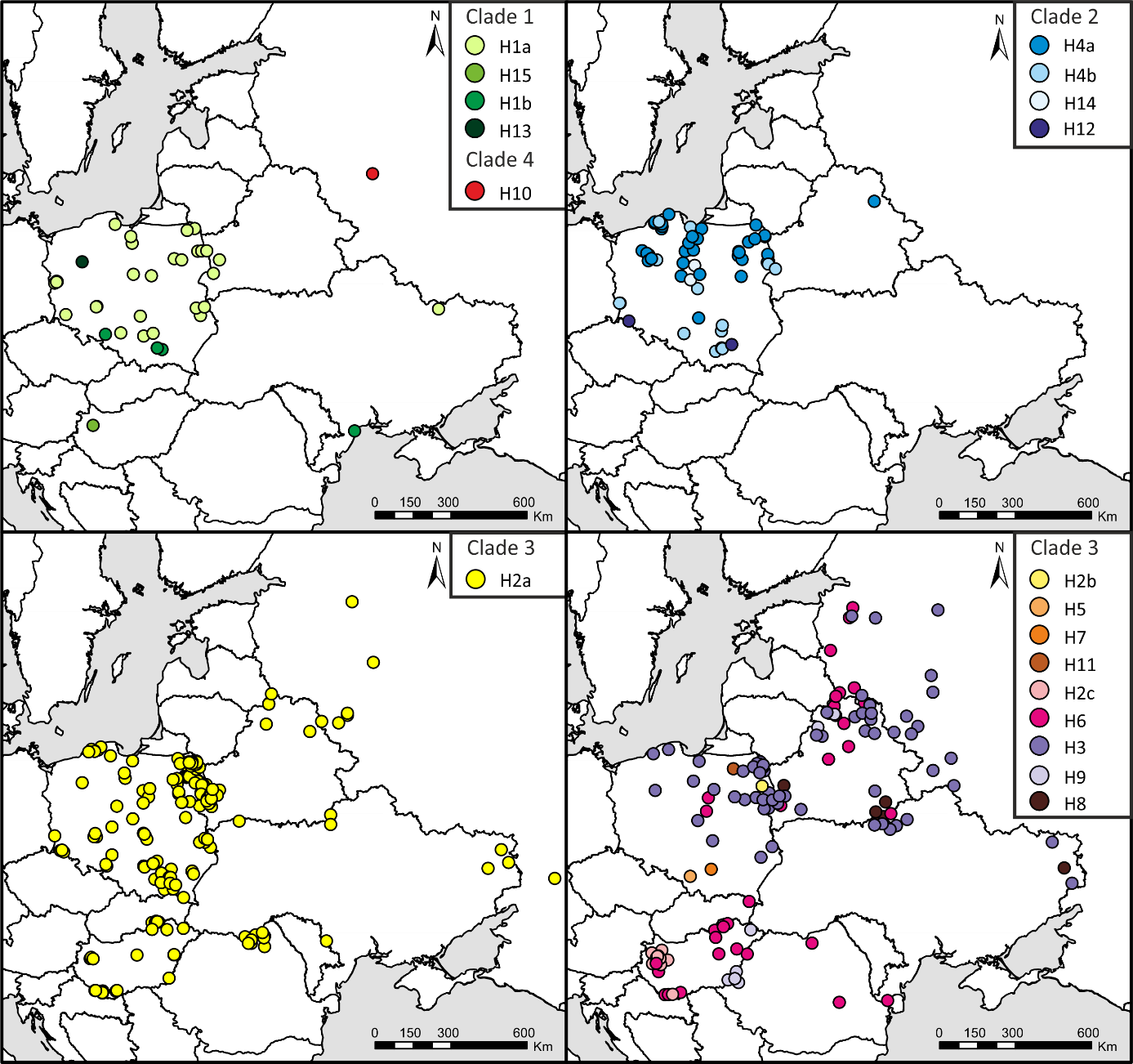


Fig. S4. Distribution of D-loop haplotypes of wild boar obtained in this study.


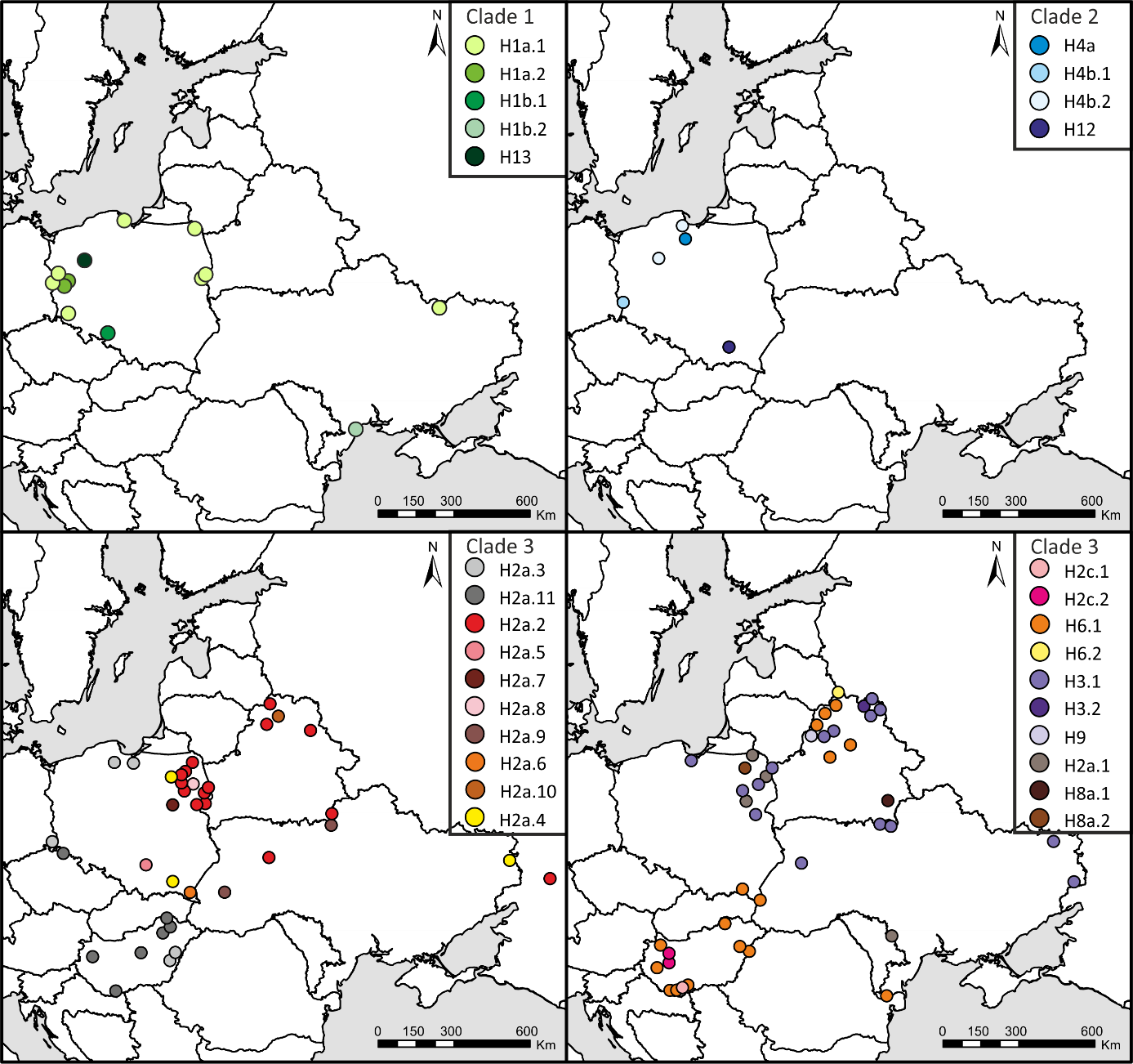


Fig. S5. Distribution of mitogenome haplotypes of wild boar obtained in this study.

**A**

**B**

**C**


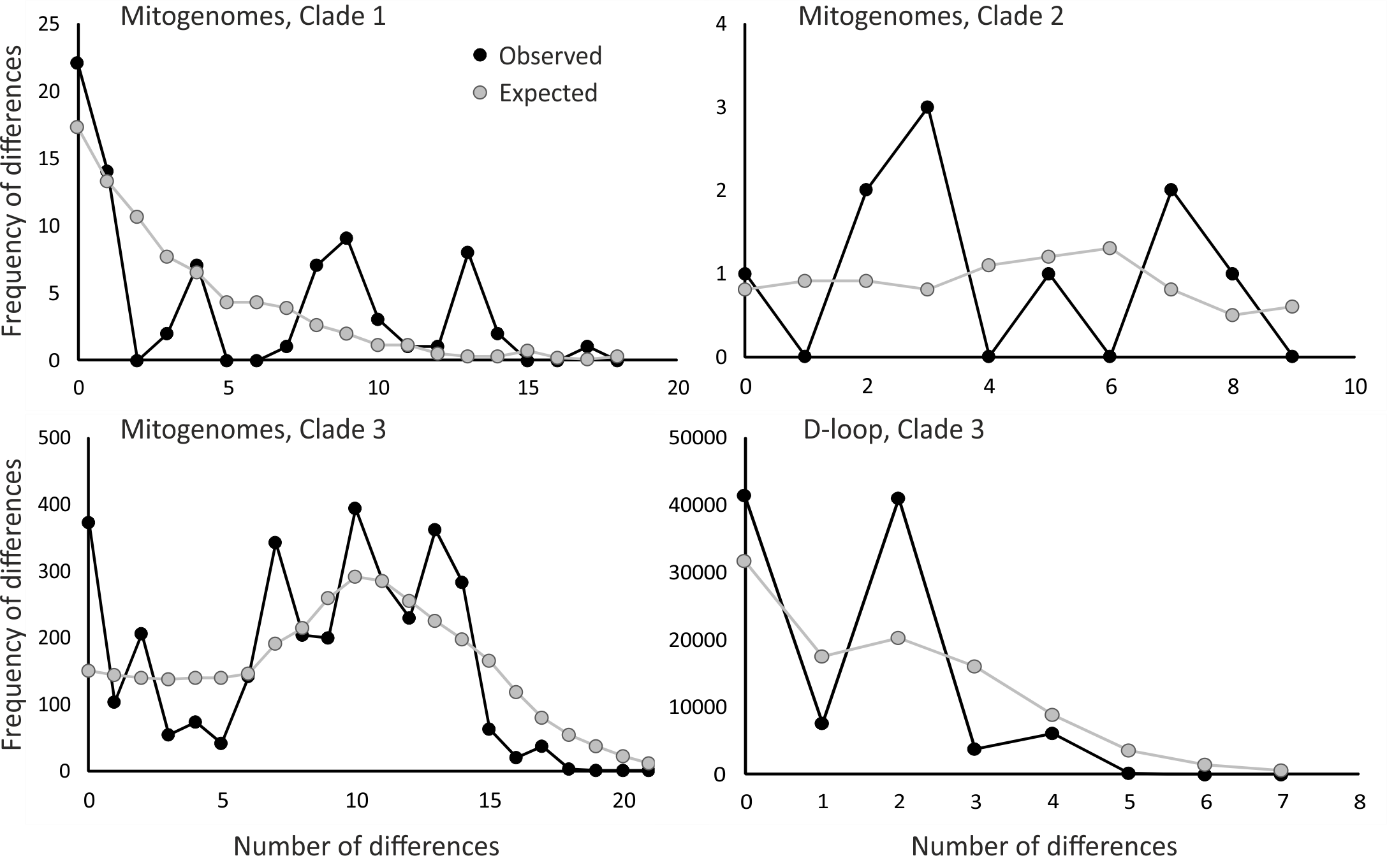


Fig. S6. Observed and expected mismatch distributions for three mitogenomic clades (upper and lower left panels) and the D-loop clade 3 (lower right panel, k – means thousand). The results for analyses of D-loop clades 1 and 2 not shown as expected and observed mismatch distributions did not converge after 2000 steps.


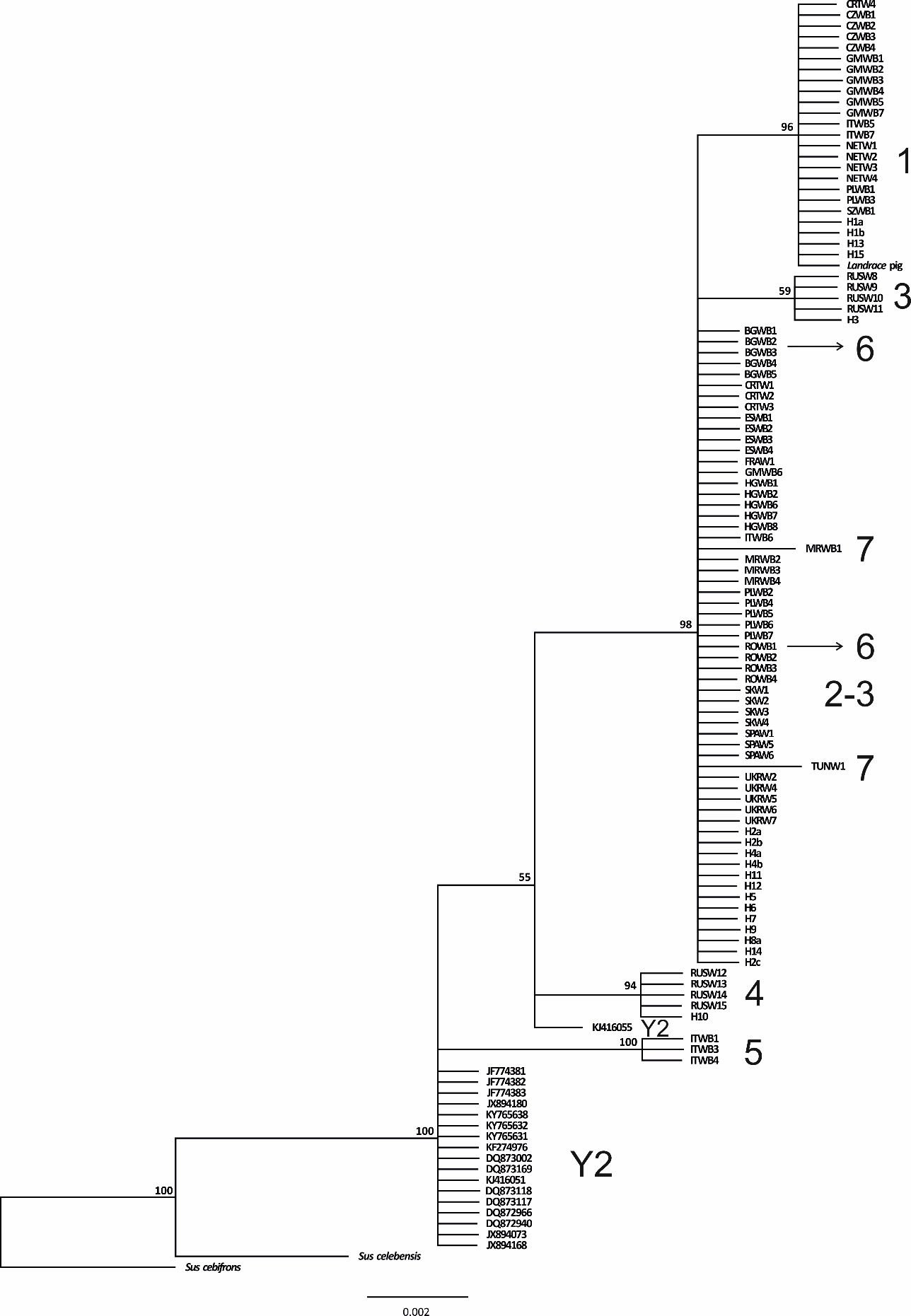


Fig. S7. The Bayesian phylogenetic tree (MCMC = 1 000 000) of D-loop sequences (75 bp) of wild boar *Sus scrofa* obtained in this study and from Gene Bank (extinct Clade Y2, *Sus cebifrons*, *S. celebensis*, accession numbers provided). Numbers of clades according to haplotype assignment based on mitogenome analyses presented in Fig. 2.


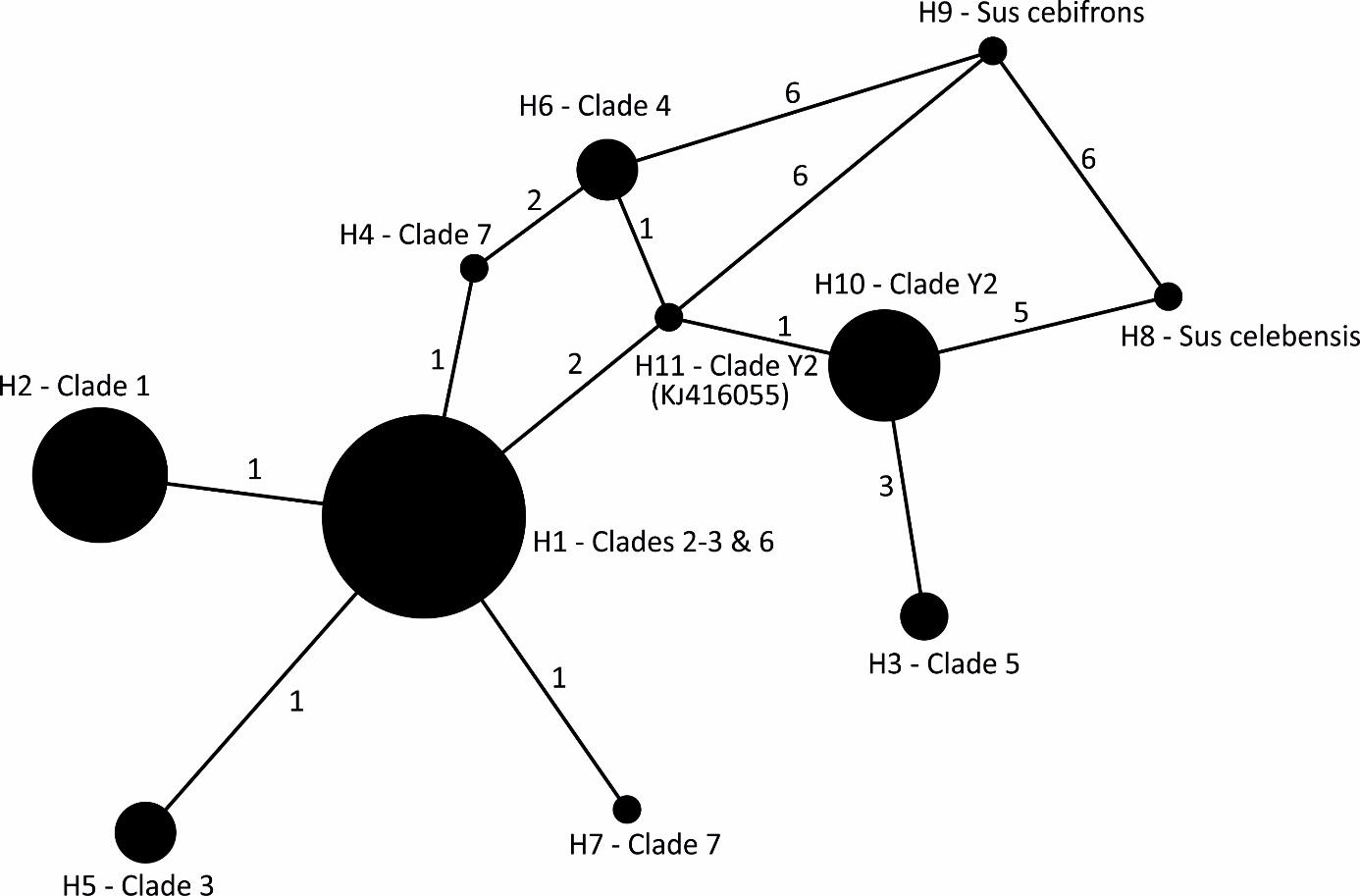


Fig. S8. Haplotype network of D-loop sequences (75 bp) of wild boar (*Sus scrofa*) obtained in this study (Clades 1-7) and available from GenBank (Clade Y2, *Sus cebifrons*, *S. celebensis,* accession numbers provided in Fig. S7). Numbers of clades as in Figs 2 and S1. Numbers on branches represent mutation steps between sequences (if > 1). The size of circle corresponds to the number of sequences with a given haplotype.

Table S1. List of sampling localities with the number of specimens and years of sampling with their assignment to the mtDNA haplotypes, mtDNA clades and Geneland populations (attached separately in the Excel format).

Table S2. Genetic diversity of wild boar populations in Central and Eastern Europe based on analyses of mitogenomic sequences; n – number of individuals, h – number of haplotypes, H_d_ – haplotype diversity, π × 10^-3^ – nucleotide diversity, *B* – haplotype diversity index, S – number of polymorphic sites, Tajima`s D, Fu`s Fs – neutrality tests. Values of neutrality tests were not significant.

| Population | n | h | Haplotypes | H_d_ | *B* |  | π | S | Tajima`s D | Fu`s Fs |
| --- | --- | --- | --- | --- | --- | --- | --- | --- | --- | --- |
| POL-N | 10 | 6 | H1a.1, H2a.3, H3.1, H4a, H4b.2, H13 | 0.84 | 4.17 |  | 0.6 | 31 | -0.206 | 2.268 |
| POL-S | 6 | 5 | H2a.4, H2a.5, H2a.6, H6.1, H12 | 0.93 | 4.5 |  | 0.6 | 25 | -0.738 | 0.762 |
| POL-W | 9 | 6 | H1a.1, H1a.2,  H1b.1, H2a.3,  H2a.11, H4b.1, | 0.89 | 4.76 |  | 0.6 | 27 | 0.033 | 1.567 |
| POL/BEL | 25 | 8 | H1a.1, H2a.1,  H2a.2, H2a.4, H2a.7, H2a.8,  H3.1, H8a.2, | 0.78 | 3.93 |  | 0.5 | 31 | -0.284 | 3.250 |
| BEL-N | 18 | 7 | H2a.2, H2a.9,  H3.1, H3.2, H6.1, H6.2, H9 | 0.83 | 4.63 |  | 0.4 | 18 | 1.381 | 2.596 |
| BEL-S | 5 | 4 | H2a.2, H2a.8, H3.1, H8a.1 | 0.90 | 3.57 |  | 0.5 | 18 | 0.307 | 1.432 |
| UKR-E | 5 | 4 | H1a.1, H2a.2, H2a.4, H3.1 | 0.90 | 3.57 |  | 0.6 | 20 | 0.000 | 1.537 |
| UKR-S | 3 | 3 | H1b.2, H2a.1, H6.1 | 1.00 | 3.00 |  | 1.0 | 25 | - | 1.676 |
| HUN | 20 | 5 | H2a.3, H2a.10,  H2c.1, H2c.2,  H6.1 | 0.73 | 3.28 |  | 0.4 | 20 | 1.223 | 6.169 |
| Total | 101 | 29 |  | 0.92 | 10.73 |  | 0.6 | 79 | -1.151 | -1.830 |

Table S3. Genetic diversity of wild boar populations in Central and Eastern Europe based on analyses of mtDNA D-loop sequences; Pop – population number (see Fig. 1), n – number of individuals, h – number of haplotypes, H_d_ – haplotype diversity, *B* – haplotype diversity index, π × 10^-3^ – nucleotide diversity, S – number of polymorphic sites, Tajima`s D, Fu`s Fs – neutrality tests,. Values of neutrality tests were not statistically significant (p > 0.05).

| Pop | n | h | Haplotypes | H_d_ | *B* |  | π | S | Tajima`s D | Fu`s Fs | |
| --- | --- | --- | --- | --- | --- | --- | --- | --- | --- | --- | --- |
| 1 | 34 | 5 | H1a, H2a, H3, H4a, H4b | 0.72 | 3.26 |  | 1.8 | 6 | 1.254 | | 1.733 |
| 2 | 10 | 5 | H2a, H3, H4a, H4b, H13 | 0.67 | 2.50 |  | 2.0 | 8 | -0.653 | | - 0.188 |
| 3 | 12 | 4 | H1a, H2a, H4b, H12 | 0.74 | 3.13 |  | 1.4 | 5 | 0.058 | | 0.518 |
| 4 | 20 | 7 | H1a, H2a, H3, H4a, H4b, H6, H14 | 0.86 | 5.55 |  | 1.9 | 7 | 0.501 | | -0.816 |
| 5 | 14 | 5 | H1a, H1b, H2a, H4b, H5 | 0.73 | 3.05 |  | 1.2 | 6 | -0.934 | | -0.806 |
| 6 | 19 | 6 | H1a, H2a, H3, H4a, H4b, H7 | 0.66 | 2.61 |  | 1.1 | 6 | -0.921 | | -1.734 |
| 7 | 31 | 5 | H1b, H2a, H4b, H6, H12 | 0.63 | 2.56 |  | 1.3 | 6 | 0.007 | | 0.514 |
| 8 | 16 | 3 | H1a, H2a, H3 | 0.51 | 1.91 |  | 0.7 | 3 | -0.360 | | 0.501 |
| 9 | 217 | 9 | H1a, H2a, H2b, H3, H4a, H4b, H6, H8a, H11 | 0.51 | 2.05 |  | 1.0 | 11 | -0.875 | | -1.386 |
| 10 | 7 | 3 | H2a, H3, H6 | 0.71 | 2.58 |  | 2.0 | 4 | 1.912 | | 1.598 |
| 11 | 53 | 6 | H2a, H3, H4a, H6, H9, H10 | 0.70 | 3.21 |  | 2.1 | 20 | -1.410 | | 1.992 |
| 12 | 17 | 4 | H2a, H3, H6, H8a | 0.62 | 2.39 |  | 1.7 | 6 | 0.481 | | 1.562 |
| 13 | 9 | 4 | H1a, H2a, H3, H8a | 0.75 | 3.0 |  | 1.4 | 5 | -0.398 | | 0.009 |
| 14 | 15 | 3 | H1b, H2a, H6 | 0.45 | 1.72 |  | 0.8 | 4 | -0.724 | | 0.823 |
| 15 | 74 | 5 | H2a, H2c, H6, H9, H15 | 0.69 | 3.01 |  | 1.2 | 6 | 0.346 | | 1.308 |
| Total | 548 | 19 |  | 0.71 | 3.41 |  | 1.5 | 29 | -1.503 | | -5.051 |

Table S4. F_ST_ values between pairs of 9 wild boar populations in Central and Eastern Europe based on mitogenomic sequences. In bold – statistical significance at p ≤ 0.01 after Bonferroni correction.

| Pop | POL-N | POL-W | POL-S | POL-BEL | BEL-N | BEL-S | UKR-E | UKR-S |
| --- | --- | --- | --- | --- | --- | --- | --- | --- |
| POL-W | 0.140 |  |  |  |  |  |  |  |
| POL-S | 0.048 | **0.290** |  |  |  |  |  |  |
| POL-BEL | 0.071 | **0.267** | 0.097 |  |  |  |  |  |
| BEL-N | 0.152 | **0.387** | 0.071 | **0.103** |  |  |  |  |
| BEL-S | 0.069 | **0.304** | 0.103 | -0.032 | -0.001 |  |  |  |
| UKR-E | 0.009 | 0.152 | 0.087 | -0.022 | 0.009 | -0.127 |  |  |
| UKR-S | 0.051 | 0.144 | 0.008 | 0.104 | 0.164 | 0.045 | 0.030 |  |
| HUN | **0.207** | **0.405** | -0.009 | **0.200** | **0.163** | **0.237** | 0.230 | 0.180 |

Table S5. F_ST_ values between pairs of 15 wild boar populations in Central and Eastern Europe, based on D-loop sequences. Statistical significance at p ≤ 0.01 after Bonferroni correction in bold. See Fig. 1 for locations of populations.

| Pop | 1 | 2 | 3 | 4 | 5 | 6 | 7 | 8 | 9 | 10 | 11 | 12 | 13 | 14 |
| --- | --- | --- | --- | --- | --- | --- | --- | --- | --- | --- | --- | --- | --- | --- |
| 2 | 0.012 |  |  |  |  |  |  |  |  |  |  |  |  |  |
| 3 | 0.122 | **0.272** |  |  |  |  |  |  |  |  |  |  |  |  |
| 4 | -0.001 | 0.096 | 0.059 |  |  |  |  |  |  |  |  |  |  |  |
| 5 | **0.162** | **0.344** | -0.041 | 0.088 |  |  |  |  |  |  |  |  |  |  |
| 6 | 0.138 | **0.346** | -0.003 | 0.059 | -0.034 |  |  |  |  |  |  |  |  |  |
| 7 | **0.098** | **0.276** | 0.010 | 0.035 | -0.009 | -0.017 |  |  |  |  |  |  |  |  |
| 8 | **0.268** | **0.498** | 0.105 | **0.191** | 0.023 | 0.023 | **0.095** |  |  |  |  |  |  |  |
| 9 | **0.271** | **0.481** | **0.154** | **0.183** | 0.078 | 0.033 | **0.098** | 0.004 |  |  |  |  |  |  |
| 10 | **0.231** | **0.337** | **0.246** | 0.104 | 0.233 | **0.220** | **0.210** | **0.275** | **0.216** |  |  |  |  |  |
| 11 | **0.236** | **0.347** | **0.211** | **0.136** | **0.179** | **0.153** | **0.184** | **0.139** | **0.155** | -0.064 |  |  |  |  |
| 12 | **0.346** | **0.460** | **0.337** | **0.267** | **0.317** | **0.296** | **0.338** | **0.257** | **0.240** | 0.096 | 0.062 |  |  |  |
| 13 | **0.247** | **0.406** | **0.159** | **0.156** | 0.116 | 0.096 | **0.161** | 0.020 | 0.010 | 0.084 | 0.033 | 0.009 |  |  |
| 14 | 0.206 | **0.419** | 0.099 | 0.118 | 0.010 | 0.012 | 0.003 | **0.079** | 0.070 | 0.200 | **0.142** | **0.331** | 0.157 |  |
| 15 | **0.218** | **0.380** | **0.179** | **0.126** | **0.120** | **0.114** | 0.069 | **0.185** | **0.183** | 0.127 | **0.166** | **0.362** | **0.224** | 0.017 |

Table S6. Genetic diversity parameters calculated for genetic groups indicated by the Geneland software for D-loop and mitogenomic sequences. Hd – haplotype diversity, π – nucleotide diversity, B - haplotype diversity index.

| Genetic groups | n | h | H_d_ | π | B |
| --- | --- | --- | --- | --- | --- |
| D-loop sequences | | | | | |
| GI | 373 | 15 | 0.64 | 0.001 | 2.74 |
| GII | 153 | 9 | 0.75 | 0.001 | 3.84 |
| GIII | 22 | 5 | 0.64 | 0.002 | 2.57 |
| D-loop sequences from Poland | | | | | |
| GPI | 58 | 7 | 0.77 | 0.002 | 4.06 |
| GPII | 279 | 12 | 0.58 | 0.001 | 2.34 |
| Mitogenomic sequences | | | | | |
| GmI | 18 | 9 | 0.89 | 0.0007 | 5.32 |
| GmII | 28 | 10 | 0.79 | 0.0005 | 4.36 |
| GmIII | 55 | 15 | 0.84 | 0.001 | 5.08 |

Table S7. Fst values among the mitogenomic groups indicated by the Geneland software;

*** p < 0.001.

|  | GmI | GmII |
| --- | --- | --- |
| GmII | 0.22*** | - |
| GmIII | 0.14*** | 0.14*** |

Table S8. Fst values calculated among the D-loop groups indicated by the Geneland software; *** p < 0.001. Fst between

the two groups in Poland only (see insert map in Fig. 6) was 0.23, p < 0.001.

|  | GI | GII |
| --- | --- | --- |
| GII | 0.08*** | - |
| GIII | 0.18*** | 0.16*** |

Table S9. List of the primers used for amplification of full mitogenome of 101 wild boar from Central and Eastern Europe. The first two primers after Kijas and Anderson (2001), all other primers after Jiang et al. (2008).

| Primer | Sequence (5’-3’) |
| --- | --- |
| SSmtDNApm33F | ATACCAATCACTAGCATCATCG |
| SSmtDNApm34R | GAGTTCCATGAAGTCCAGCTAC |
| 1F | CTTACTTCAGGACCATCTCA |
| 1B | GGTTGAGCAAGGCGTTAT |
| 2F | GTGAGAATGCCCTCCAGATC |
| 2B | CCCTTACGGTACTATCTCTATAGCG |
| 3F | GGATTACCAAAGCATAGC |
| 3B | TGATTGGAAGTAAGAGACAG |
| 4F | CTGCCCAGTGACACCAGT |
| 4B | CCTGGTCGTAGGGGTTCT |
| 5F | CCCTAGTAGAACGAAAAGTG |
| 5B | TAGGGAGTGAGATGTGTC |
| 6F | CACGATTCCGATACGACC |
| 6B | GCAGGCCTCCTATTGAG |
| 7F | CATACTCATCAATCGCAC |
| 7B | ACAGTTCATCCAGTACCC |
| 8F | TGCCTGAGCAGGAATAGTG |
| 8B | CATGCTTGGTTGAGTGTGTAC |
| 9F | AGTCGCACACTTCCACTA |
| 9B | GTGCAGTACGTCTTCAGA |
| 10F | CTTAAACCTGGAGAAATACG |
| 10B | TAGGGCTACTGGTTGAATAA |
| 11F | CCCCCTATGATCAGCAACCGT |
| 11B | GCAACCCGTGAAACCCTG |
| 12F | AAGGCCACCACACATCAG |
| 12B | GGTAGGGGAAGGAGAAGG |
| 13F | CCTGATACTGACACTTCGTAG |
| 13B | TAAGGGGGAGGAGT |
| 14F | TCCGACTCACTATCAGCA |
| 14B | TTAGTATGTAGAGAGAGTAGAGGG |
| 15F | AGCCCTACCCCCATCCATC |
| 15B | AATTCCTACGCCTTCTCATCC |
| 16F | CTACGCCTTCACTCTCAGC |
| 16B | CTCAGGCGTTTGTGTAGGA |
| 17F | TCTCACAGGATTCTACTC |
| 17B | TACTACTGCTATGGCTACTG |
| 18F | CTTCCTCTTCCTTCAACGCA |
| 18B | AGCCCCCTCAGATTCATTC |
| 19F | GTCCTGCCCTGAGGACAA |
| 19B | GGTGCTGATGGCGGAGTT |
